# Novelty and Convergence in Adaptation to Whole Genome Duplication

**DOI:** 10.1093/molbev/msab096

**Published:** 2021-03-30

**Authors:** Magdalena Bohutínská, Mark Alston, Patrick Monnahan, Terezie Mandáková, Sian Bray, Pirita Paajanen, Filip Kolář, Levi Yant

**Affiliations:** 1 Department of Botany, Faculty of Science, Charles University, Prague, Czech Republic; 2 Institute of Botany, The Czech Academy of Sciences, Průhonice, Czech Republic; 3 Department of Cell and Developmental Biology, John Innes Centre, Norwich Research Park, Norwich, United Kingdom; 4 CEITEC—Central European Institute of Technology, and Faculty of Science, Masaryk University, Kamenice, Czech Republic; 5 Future Food Beacon of Excellence, University of Nottingham, Nottingham, United Kingdom; 6 School of Biosciences, University of Nottingham, Nottingham, United Kingdom; 7 Natural History Museum, University of Oslo, Oslo, Norway; 8 School of Life Sciences, University of Nottingham, Nottingham, United Kingdom

**Keywords:** polyploidy, convergence, genome duplication, adaptation

## Abstract

Whole genome duplication (WGD) can promote adaptation but is disruptive to conserved processes, especially meiosis. Studies in *Arabidopsis arenosa* revealed a coordinated evolutionary response to WGD involving interacting proteins controlling meiotic crossovers, which are minimized in an autotetraploid (within-species polyploid) to avoid missegregation. Here, we test whether this surprising flexibility of a conserved essential process, meiosis, is recapitulated in an independent WGD system, *Cardamine amara*, 17 My diverged from *A. arenosa*. We assess meiotic stability and perform population-based scans for positive selection, contrasting the genomic response to WGD in *C. amara* with that of *A. arenosa*. We found in *C. amara* the strongest selection signals at genes with predicted functions thought important to adaptation to WGD: meiosis, chromosome remodeling, cell cycle, and ion transport. However, genomic responses to WGD in the two species differ: minimal ortholog-level convergence emerged, with none of the meiosis genes found in *A. arenosa* exhibiting strong signal in *C. amara*. This is consistent with our observations of lower meiotic stability and occasional clonal spreading in diploid *C. amara*, suggesting that nascent *C. amara* autotetraploid lineages were preadapted by their diploid lifestyle to survive while enduring reduced meiotic fidelity. However, in contrast to a lack of ortholog convergence, we see process-level and network convergence in DNA management, chromosome organization, stress signaling, and ion homeostasis processes. This gives the first insight into the salient adaptations required to meet the challenges of a WGD state and shows that autopolyploids can utilize multiple evolutionary trajectories to adapt to WGD.

## Introduction

Whole genome duplication (WGD) is both a massive mutation and a powerful force in evolution. The opportunities and challenges presented by WGD emerge immediately, realized in a single generation. As such, WGD comes as a shock to the system. Autopolyploids, formed by within-species WGD (without hybridization), result from the chance encounter of unreduced gametes (with diverse underlying factors, see Mason and Pires 2015). Thus, they typically harbor four full haploid genomes that are similar in all pairwise combinations, resulting in a lack of pairing partner preferences at meiosis. This, combined with multiple crossover events per chromosome pair, can result in multivalents among three or more homologs at anaphase, increasing the likelihood of missegregation or chromosome breakage, leading to aneuploidy (Bomblies and Madlung 2014; Bomblies et al. 2016). Beyond this, WGD presents a suddenly transformed intracellular landscape to the conserved workings of the cell, such as altered ion homeostasis and a host of nucleotypic factors related to cell size, volume, and cell cycle progression (Chao et al. 2013; Yant and Bomblies 2015; Doyle and Coate 2019; Bomblies 2020). 

Despite this, some lineages survive this early trauma and successfully speciate, with direct empirical evidence of the increased adaptability of autopolyploid lineages from in vitro evolutionary competition experiments in yeast (Selmecki et al. 2015). With increased ploidy, genetic variability can be maintained in a masked state, with evidence of young WGD lineages further recruiting diverse alleles by gene flow across ploidies, and indeed, species (Arnold et al. 2016; Marburger et al. 2019; Monnahan et al. 2019). At the genomic level, recent detailed understanding of gene flow following WGD supports the idea that WGD can cause the breakdown of species barriers present in diploids. Evidence for this has come from both plants (*Arabidopsis arenosa/Arabidopsis lyrata* [Schmickl and Koch 2011]) and animals (the frog genus *Neobatrachus* [Novikova et al. 2020], reviewed in Schmickl and Yant 2021). In both examples WGD led to niche expansion (Molina-Henao and Hopkins 2019; Novikova et al. 2020) and the invasion of particularly challenging environments relative to the diploid: in the case of polyploid frogs, the desert (Novikova et al. 2020) and polyploid *A. arenosa*, metal-contaminated mines and serpentine barrens (Arnold et al. 2016; Preite et al. 2019; Konečná et al. 2021). Thus, although clear challenges must be overcome to function as a polyploid (Bomblies et al. 2015; Yant and Bomblies 2015; Baduel, Hunter, et al. 2018), novel population genomic and ecological opportunities await a lineage that successfully adapts to a WGD state (Yant and Bomblies 2015; Baduel, Hunter, et al. 2018).

The functional and genomic basis for adaptation to WGD has been closely investigated in *A. arenosa*, which exists as both diploid and young autotetraploid lineages (∼20,000 generations old; Arnold *et al.* 2015; Kolář et al. 2016). Population genomic scans for selection using a diversity of metrics have shown the strongest signals of positive selection following WGD in *A. arenosa* as sharp, single-gene peaks over 10 genes that physically and functionally interact to control meiotic chromosome crossovers (Hollister et al. 2012; Yant et al. 2013; Bohutínská et al. 2021a). During early meiotic chromosome crossover formation in an autotetraploid, the four copies of each chromosome are impossible to distinguish. Thus, crossovers can occur haphazardly in any pairwise manner. If more than one crossover per chromosome pair is allowed to occur, multivalent associations can result, leading to aneuploidy at anaphase. Thus a reduction in the number of meiotic crossovers to one per chromosome pair stands as the leading candidate process mediating adaptation to WGD (Bomblies et al. 2016). In the young *A. arenosa* autotetraploids harboring these derived alleles, we observed a decrease in meiotic crossover number as well as fewer multivalents relative to synthetic autopolyploids with ancestral-like diploid alleles (Yant et al. 2013). Recent work found that the closely related sister species *A. lyrata*, which contains a younger autotetraploid lineage, also harbors many of the same selected alleles discovered in *A. arenosa* (Marburger et al. 2019)*.* Moreover, from a joint population genomic analysis of both species across an established natural hybrid zone between *A. arenosa* and *A. lyrata*, clear gene sized signals of directional adaptive gene flow and positive selection emerge precisely at these alleles specifically between the two tetraploids (Marburger et al. 2019; Seear et al. 2020), indicating that *A. lyrata* and *A. arenosa* WGD stabilization events are not fully independent. Among these candidate adaptive alleles at least one has been functionally shown to modulate adaptive decreases in crossover numbers (Morgan et al. 2020; Seear et al. 2020),

Here, we use an independent system, approximately 17 My diverged from both *A. arenosa* and *A. lyrata* (Huang et al. 2020), to test the hypothesis that this solution of meiosis gene evolution is repeated, and if not, whether changes in other genes from analogous processes are associated with adaptation to WGD. Given the clear results in *A. arenosa* and *A. lyrata*, we hypothesized that the adaptive trajectories which are available to mediate adaptation to a WGD state are constrained, leading to repeated selection of the same suite of meiosis genes. Such a result would offer a striking case of convergent evolution in core cellular processes. To test this hypothesis, we take advantage of a well-characterized model, *Cardamine amara* (Brassicaceae, tribe Cardamineae). A large-scale cytotyping survey of over approximately 3,300 individuals in 302 populations and genetic analysis detail the demographic relationships of this diploid/tetraploid complex in the Eastern and Central Alps (Zozomová-Lihová et al. 2015). Comparison of genotyping results of this study with simulations indicates a single autotetraploid origin. Importantly, *C. amara* is a perennial herb harboring a high level of genetic diversity and shares with *A. arenosa* a similar distribution range and evolutionary history, with a likely single geographic origin, followed by autotetraploid expansion associated with glacial oscillations (Marhold et al. 2002; Zozomová-Lihová et al. 2015).

To test our hypothesis that gene-level evolutionary convergence is likely following WGD, we performed genome scans for positive selection in both *C. amara* and *A. arenosa*, contrasting natural autotetraploid and diploid populations in both species. Because there was no reference genome available for *C. amara*, we first generated a novel quality reference. We then tested for convergence in the evolutionary response to WGD at the level of the ortholog, process, and network in a sampling of 100 *C. amara* and 120 *A. arenosa* individuals from well-assessed ranges (Arnold et al. 2015; Zozomová-Lihová et al. 2015; Kolář et al. 2016; Monnahan et al. 2019). Overall, we found that the evolutionary response to WGD in *C. amara* is very different to that of *A. arenosa*, with none of the orthologous genes that control meiotic chromosome crossovers in *A. arenosa* under strong selection in *C. amara*. In contrast, we find a clear signal of process-level convergence in core pathways controlling DNA management and chromosome organization.

## Results and Discussion

### Reference Genome, Population Selection, Sampling, and Genetic Structure

**Fig. 1.**
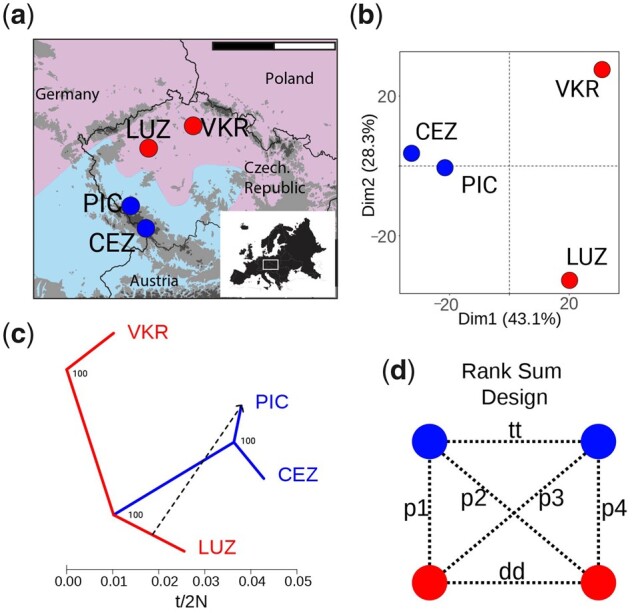
Sampling and population structure of *Cardamine amara*. (*a*) Locations of diploid (red) and autotetraploid (blue) *C. amara* populations sampled. Scale bar corresponds to 200 km; shaded area represents each cytotype range in Zozomová-Lihová et al. (2015). (*b*) Population differentiation represented by Principal Component Analysis of approximately 124,000 4-fold degenerate SNPs. (*c*) Phylogenetic relationships and migration events between populations inferred by TreeMix analysis. *X*-axis shows the drift estimation, corresponding to the number of generations separating the two populations (*t*), and effective population size (*N*) (Pickrell and Pritchard 2012). Node labels show bootstrap support, and the arrow indicates the most likely migration event (migration weight, which can be interpreted as a moderate degree of admixture = 0.18, similar to *Arabidopsis arenosa*, shown in supplementary fig. 1, Supplementary Material online). Additional migration events did not improve the model likelihood. (*d*) Rank Sum design used in divergence scans to minimize potential bias of population-specific divergence. p1–p4 represent the between-ploidy contrasts used for the rank sum calculations. dd and tt represent within-ploidy contrasts used to subtract signal of local population history within each cytotype.

Because *C. amara* is approximately 17 My diverged from *A. arenosa* (Huang et al. 2020), using the same reference genome for mapping reads of both species would result in unacceptably low mapping efficiencies and missing data. We therefore first generated a novel reference genome for *C. amara* (N50 = 1.82 Mb, 95% complete BUSCOs; see Materials and Methods). We then resequenced in triplicate four populations of contrasting ploidy, sampling 100 individuals: two diploid (LUZ, VRK) and two autotetraploid (CEZ, PIC; fig. 1*a*;supplementary table 1, Supplementary Material online). We chose these populations based on a comprehensive cytological and demographic survey of approximately 3,300 *C. amara* samples throughout the Czech Republic (Zozomová-Lihová et al. 2015). Sampled plants were spaced at least 3 m apart, as this distance was sufficient to avoid resampling of identical clones in that study. We chose populations to represent core areas of each cytotype, away from potential hybrid zones and distant from any triploid-containing populations based on (Zozomová-Lihová et al. 2015). Further, we performed flow cytometry on every sample sequenced to verify expected ploidy.

To obtain robust population allele frequency (AF) estimates across genomes, we performed a replicated pooled sequencing approach. From every population we pooled DNA from 25 individuals and generated on average 31 million reads per sample (for all samples we generated triplicate DNA preps, pooling, and sequencing to control for potential sampling error: details in Materials and Methods) and mapped reads to our new *C. amara* assembly (mean coverage per population = 86, supplementary table 2, Supplementary Material online). After mapping, variant calling and quality filtration, we obtained a final data set of 2,477,517 SNPs.

The first PCA axis dominantly explained 43% of variation (fig. 1*b*) and was consistent with differentiation primarily by geographic distribution or ploidy (which coincide), followed by differentiation between the two diploid populations from each other (second axis explaining 28% of variation). The two autotetraploid populations clustered together in the TreeMix graph (fig. 1*c*) and had the lowest genetic differentiation of all contrasts (*F*_st_ = 0.04, mean AF difference = 0.06, table 1) and lacked any fixed SNP difference whatsoever (table 1). This high genetic similarity and spatial arrangement (the populations represent part of a continuous range of the autotetraploid cytotype), suggest that both autotetraploid populations represent the outcome of a single polyploidization event, in line with previous assessments (Marhold et al. 2002; Zozomová-Lihová et al. 2015), although multiple tetraploid origins cannot be ruled out. The absence of individual-level genotype information did not allow for exact dating, but nearly identical levels of interploidy divergence in both *C. amara* and *A. arenosa* (average *F*_st_ between diploids and autotetraploids = 0.10 and 0.11, respectively) and comparable drift estimates in TreeMix (supplementary fig. 1, Supplementary Material online), suggested that the polyploidization may be roughly the same age (table 1). Supporting this, both WGD events were estimated to correspond with the end of the last European glaciation (Marhold et al. 2002; Arnold et al. 2015; Zozomová-Lihová et al. 2015).

**Table 1.** Measures of Genome-wide Differentiation between *Cardamine amara* and *Arabidopsis arenosa* Populations.

Populations	Ploidies	Mean AFD	Fixed Diffs	Mean *F*_st_	No. of SNPs
PIC–VKR	4× – 2×	0.09	30	0.09	2,326,315
PIC–LUZ	4× – 2×	0.09	2	0.08	2,314,229
CEZ–VKR	4× – 2×	0.11	120	0.12	2,333,538
CEZ–LUZ	4× – 2×	0.11	86	0.11	2,335,004
CEZ–PIC	4× – 4×	0.06	0	0.04	2,297,229
LUZ–VKR	2× – 2×	0.10	6	0.09	2,018,892
*A. arenosa* tetraploids–*A. arenosa* diploids	4× – 2×	0.05	21	0.11	7,106,848

Note.—Differentiation metrics shown are genome-wide mean allele frequency difference between populations (Mean AFD), the number of fixed differences (Fixed diffs) and mean *F*_st_ (Nei 1972). In the case of *A. arenosa*, *F*_st_ in diploids is calculated as a mean over all pairwise *F*_st_ measurements between the five previously characterized diploid lineages (Monnahan et al. 2019).

### Selection Specifically Associated with WGD in *C. amara*

To minimize false positives due to local population history we leveraged a quartet-based design (Vijay et al. 2016), consisting of two diploid and two autotetraploid populations (details in Materials and Methods). The mean number of SNPs per population contrast was 2,270,868 (table 1). We calculated *F*_st_ for 1-kb windows with a minimum 20 SNPs for all six possible population contrasts (fig. 1*d*), and ranked windows based on *F*_st_ values. To focus on WGD-associated adaptation, we first assigned ranks to each window based on the *F*_st_ values in each of four possible pairwise diploid–autotetraploid contrasts and identified windows in the top 1% outliers of the resultant combined rank sum (fig. 1*d*, contrasts p1–p4). We then excluded any window which was also present in the top 1% *F*_st_ outliers in diploid–diploid or autotetraploid–autotetraploid population contrasts to avoid misattribution caused by local population history (fig. 1*c*, contrasts tt and dd). By this approach, we identified 440 windows that intersected 229 gene coding loci (supplementary data set 1, Supplementary Material online; termed WGD adaptation candidates below). To control for possible biases due to suboptimal window size selection, we recalculated *F*_st_ on a SNP-by-SNP basis, considering genes with 5 or more SNPs. This approach resulted in the comparable candidate list to the window-based analysis (see Materials and Methods). Larger windows (50 kb) failed to detect peaks of divergence.

Among these 229 gene coding loci, a Gene Ontology (GO) term analysis yielded 22 significantly enriched biological processes (Fisher’s exact test with conservative “elim” method, *P* < 0.05, supplementary table 3, Supplementary Material online). To further control for false positives and refine this candidate list to putatively functional candidates, we complemented these differentiation measures with a quantitative estimate that incorporates potential functional impact of encoded derived amino acid changes, following the FineMAV method (Szpak et al. 2018) (see Materials and Methods for a full description). In short, as an orthogonal complement to *F*_st_ scans above, FineMAV assigns SNPs a score based on the predicted functional consequences of resultant amino acid substitutions using Grantham scores, and amplifies these by the per-cytotype AF difference between the two amino acids (Szpak et al. 2018, Bohutínská et al. 2021a). This allowed us to focus on radical amino acid changes driven to high frequency specifically in the autotetraploids. From our 229 *F*_st_ window-based WGD adaptation candidates, 120 contained at least one 1% FineMAV outlier amino acid substitution (supplementary data sets 1 and 2, Supplementary Material online).

### DNA Maintenance (Repair, Chromosome Organization) and Meiosis under Selection in *C. amara*

**Fig. 2.**
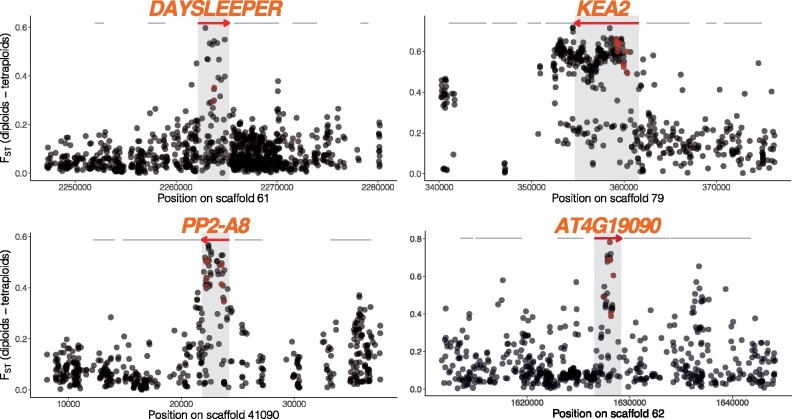
Selective sweep signatures at DNA management and ion homeostasis loci. Examples of selective sweep signatures among four candidate loci (red arrows). *X*-axis gives scaffold position in base pairs. *Y*-axis gives *F*_st_ values at single nucleotide polymorphisms (dots) between diploid and autotetraploid *Cardamine amara*. Red dots indicate FineMAV outlier SNPs. Red arrows indicate gene models overlapping top 1% *F*_st_ windows and gray lines indicate neighboring gene coding loci.

Of the 22 significantly enriched GO processes, the most enriched by far was DNA metabolic process (*P*-value = 6.50E-08, vs. 0.00021 for the next most confident enrichment), although there was also enrichment for chromosome organization and meiotic cell cycle. The 40 genes contributing to these categories showed highly localized peaks of differentiation (fig. 2), as well as 1% FineMAV outlier SNPs in coding regions (fig. 2, supplementary data sets 1 and 2, Supplementary Material online). These genes also clustered in STRING interaction networks, suggesting coevolutionary dynamics driving the observed selection signals (supplementary fig. 2, Supplementary Material online; see Materials and Methods). The largest cluster comprised of *MSH6, PDS5e, SMC2, MS5, PKL, HDA18, CRC*, and homologs of two uncharacterized, but putative DNA repair related loci *AT1G52950* and *AT3G02820* (containing SWI3 domain). *MutS Homolog 6* (*MSH6*) is a component of the postreplicative DNA mismatch repair system. It forms a heterodimer with MSH2 which binds to DNA mismatches (Culligan and Hays 2000; Wu et al. 2003), enhancing mismatch recognition. *MutS* homologs have also been shown to control crossover number in *Arabidopsis thaliana* (Lu et al. 2008). The *C. amara* ortholog of *AT1G15940* is a close homolog of *PDS5*, a protein required in fungi and animals for formation of the synaptonemal complex and sister chromatid cohesion (Panizza et al. 2000)*. Structural Maintenance Of Chromosomes 2* (*SMC2/TTN3*) is a central component of the condensin complex, which is required for segregation of homologous chromosomes at meiosis (Siddiqui et al. 2003) and stable mitosis (Liu and Meinke 1998). *PICKLE* (*PKL*) is a SWI/SWF nuclear-localized chromatin remodeling factor (Ogas et al. 1999; Shaked et al. 2006) that also has highly pleiotropic roles in osmotic stress response (Perruc et al. 2007), stomatal aperture (Kang et al. 2018), root meristem activity (Aichinger et al. 2011), and flowering time (Jing et al. 2019). Beyond this cluster, other related DNA metabolism genes among our top outliers include *DAYSLEEPER* (fig. 2), a domesticated transposase that is essential for development, first isolated as binding the *Kubox1* motif upstream of the DNA repair gene *Ku70* (Bundock and Hooykaas 2005). The complex Ku70/Ku80 regulate nonhomologous end joining (NHEJ) double-strand break repair (Tamura et al. 2002). Consistent with this, *DAYSLEEPER* mutants accumulate DNA damage (Knip 2012), but the exact role of *DAYSLEEPER* in normal DNA maintenance is not yet understood. Interesting also is the identification of *MALE-STERILE 5* (*MS5/TDM1*), which is required for cell cycle exit after meiosis II. As the name implies, MS5 mutants are male sterile, with pollen tetrads undergoing an extra round of division after meiosis II without chromosome replication (Glover et al. 1998). *MS5/TDM1* may be an APC/C component whose function is to ensure meiosis termination at the end of meiosis II (Cifuentes et al. 2016). Together, this set of DNA management loci exhibiting the strongest signals of selection points to widespread modulation of DNA repair and chromosome management following WGD in *C. amara.*

### Evolution of Stress Signaling and Ion Homeostasis Genes

The remainder of the enriched GO categories in *C. amara* revolved around a diversity of cellular processes, including stress response, protein phosphorylation, root development, ABA signaling, and ion homeostasis. The intersection of these processes was often represented by several genes. For example, two of the top 20 highest-scoring SNPs in the genome-wide FineMAV analysis reside in SNF1-related protein kinase SnRK2.9 (supplementary data set 2, Supplementary Material online). SnRKs have been implicated in osmotic stress and root development (Fujii et al. 2011; Kawa et al. 2020), and their activity also mediates the prominent roles of Clade A protein phosphatase 2C proteins in ABA and stress signaling (Cutler et al. 2010). Interesting in this respect is a strong signature of selection in *HIGHLY ABA-INDUCED PP2C GENE 1*, a clade A *PP2C* protein (supplementary data set 1, Supplementary Material online). Stress-related phosphoinositide phosphatases are represented by *SAC9*, mutants of which exhibit constitutive stress responses (Williams et al. 2005). Diverse other genes related to these categories exhibit the strongest signatures of selection, such as *PP2-A8* (Meyers et al. 2002) and *AT4G19090*, a transmembrane protein strongly expressed in young buds (Klepikova et al. 2016) (fig. 2).

Given the observed increase in potassium and dehydration stress tolerance in first generation autotetraploid *A. thaliana* (Chao et al. 2013), it is very interesting that our window-based outliers included an especially dramatic selective sweep at *K^+^ Efflux Antiporter 2* (*KEA2*, fig. 2), a K^+^ antiporter that modulates osmoregulation, ion, and pH homeostasis (Kunz et al. 2014). Recent evidence indicates that *KEA2* is important for eliciting a rapid hyperosmotic-induced Ca^2+^ response to water limitation imposed by osmotic stress (Stephan et al. 2016). The *KEA2* locus in autotetraploid *C. amara* features an exceptional ten FineMAV-outlier SNPs (fig. 2, supplementary data sets 1 and 2, Supplementary Material online), indicating that the sweep contains a run of radical amino acid changes at high AF difference between the ploidies, pointing to a potential functional change. We also detected *cation-chloride co-transporter 1* (*HAP 5*) a Na^+^, K^+^, Cl^−^ cotransporter, involved in diverse developmental processes and Cl^−^ homeostasis (Colmenero-Flores et al. 2007). These cellular processes map well onto increasingly recognized changes that occur in polyploids, most comprehensively reviewed by (Bomblies 2020).

### Limited Gene Ortholog-Level Convergence between *C. amara* and *A. arenosa*

We hypothesized that WGD imposed strong, specific selection pressures leading to convergent directional selection on the same genes or at least on different genes playing a role in the same process (ortholog- or function-level convergence, respectively) between *C. amara* and *A. arenosa*. To test for this, we complemented our *C. amara* genome scan with an analysis of *A. arenosa* divergence outliers based on an expanded sampling relative to the original *A. arenosa* genome scan studies. We selected the 80 diploid and 40 autotetraploid individuals sequenced most deeply in a recent range-wide survey (Monnahan et al. 2019, subsampling following Bohutínská et al. 2021a) of genomic variation in *A. arenosa* (mean coverage depth per individual = 18; 160 haploid genomes sampled of each ploidy), and scanned for *F*_st_ outliers in 1-kb windows, as we did for *C. amara*. We identified 696 windows among 1% *F*_st_ outliers, overlapping 452 gene-coding loci (supplementary data set 3, Supplementary Material online), recovering results similar to (Yant et al. 2013, Bohutínská et al. 2021a), including the interacting set of loci that govern meiotic chromosome crossovers, despite radically different sampling in each of the *A. arenosa* studies. From this entire list of 452 *A. arenosa* WGD adaptation candidates, only six orthologous loci were shared with our 229 *C. amara* WGD adaptation candidates (table 2). Although it is possible that these six genes may be convergently evolving in each species, this degree of overlap was not significant (*P* = 0.42, Fisher’s exact test), indicating no excess convergence at the level of orthologous genes beyond the quantity expected by chance. Re-analysis with candidate genes detected using the SNP-by-SNP divergence scan did not identify any additional convergent gene. Similarly, there was no excess overlap among genes which harbor at least one candidate FineMAV substitution (3 overlapping candidate genes out of 120 in *C. amara* and 303 in *A. arenosa*; *P* = 0.27, Fisher’s exact test). This lack of excess convergence at the ortholog level may come as a surprise given the expected shared physiological challenges attendant to WGD (Yant and Bomblies 2015; Baduel, Bray, et al. 2018; Bomblies 2020).

**Table 2.** WGD Adaptation Candidates in Both *Arabidopsis arenosa* and *Cardamine amara*.

*C. amara* ID	*Arabidopsis thaliana* ID	*A. arenosa* ID	Name	Function (TAIR)
*CAg1480*	*AT1G16460*	*AL1G28600*	MST2/RDH2	Embryo/seed development
*CAg20214*	*AT2G45120*	*AL4G44210*	C2H2-like zinc finger	Stress response
*CAg11103*	*AT3G42170*	*AL3G27110*	DAYSLEEPER	DNA repair
*CAg16465*	*AT3G62850*	*AL1G11960*	Zinc finger-like	Unknown
*CAg4024*	*AT5G05480*	*AL6G15370*	Asparagine amidase A	Growth and development
*CAg5641*	*AT5G23570*	*AL6G34840*	SGS3	Posttranscriptional gene silencing

Note.—The number of genes does not exceed random expectations for the overlap of candidate gene lists from each species, indicating a lack of gene-level convergence. Underlined genes also harbor at least one candidate FineMAV SNP in both species.

To determine whether we may have failed to detect convergent loci due to missing data or if top outliers in *A. arenosa* had few, but potentially functionally implicated, differentiated SNPs in *C. amara*, we performed a targeted search in *C. amara* for the interacting set of meiosis proteins found to exhibit the most robust signatures of selection in *A. arenosa* (Yant et al. 2013; Bohutínská et al. 2021a) (supplementary table 4, Supplementary Material online). All meiosis-related orthologs in *C. amara* that exhibit selection signatures in *A. arenosa* (13 in total) passed our data quality criteria and were included in our analyses. Only three showed any signal by FineMAV analysis: *PDS5b* harbors an unusually high three fineMAV outlier SNPs, although it is not a *F*_st_ outlier. *ASY3*, which controls crossover distribution at meiosis, has only one FineMAV outlier SNP. Finally, a regulator of endoreduplication, *CYCA2; 3*, also harbors a single FineMAV 1% outlier in *C. amara*, although it was not included in the *F*_st_ window analysis (the window overlapping it contained only 7 SNPs, below the 20 SNP minimum cut-off for inclusion in the *F*_st_ window analysis). However, these 7 SNPs exhibited high mean *F*_st_ (0.55). Thus, although we detect varying signal in these three meiosis-related genes following WGD (supplementary table 4, Supplementary Material online), we do not see widespread signals of selection in the set of interacting crossover-controlling genes that were so conspicuous in *A. arenosa* (Yant et al. 2013).

### Meiotic Stability in *C. amara*

**Fig. 3.**
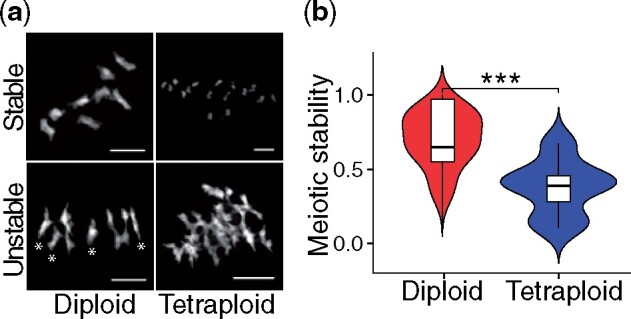
Variable meiotic stability in *Cardamine amara*. (*a*) An example of stable and unstable diploid and autotetraploid DAPI-stained meiotic chromosomes (diakinesis and metaphase I). Unstable meiosis is characterized by multivalent formation and interchromosomal connections, so we use the proportion of bivalents to multivalents as a proxy to estimate stability. In this example, the stable and unstable diploids (left panels) pictured contain 8 and 4 bivalents, respectively, whereas the stable and unstable tetraploids (right panels) show 16 and 0 bivalents, respectively. Thus all chromosomes pictured in these “Stable” examples are present as bivalents, whereas in the “Unstable” examples, only the four with asterisks (*) are bivalents, whereas the rest are mulivalents. Scale bar corresponds to 10 µm. For a complete overview of all scored chromosome spreads see supplementary figure 5, Supplementary Material online. (*b*) Distribution of meiotic stability (calculated as proportion of stable and partly stable to all scored meiotic spreads) in diploid and autotetraploid individuals of *C. amara.* *** *P* < 0.001, GLM with binomial errors.

Despite our broad overall analysis of selection in *C. amara*, as well as a targeted assessment of particular meiosis genes, we did not detect strong signal of selection in meiosis genes in *C. amara* (supplementary table 4, Supplementary Material online). The *C. amara* autotetraploid is a fertile, outcrossing, well-established lineage, but we still wondered if some contrast in meiotic behavior underlies this difference in specific loci under selection. We therefore cytologically assessed the degree of male meiotic stability in *C. amara* (fig. 3*a*). A reduction in crossover number to one per bivalent is indicated as a leading mechanism for meiotic diploidization in autopolyploids because this limits multivalent associations (which increase the propensity toward breakage and aneuploidy vs. bivalents [Cifuentes et al. 2010; Le Comber et al. 2010; Bomblies et al. 2016]), so we use proportion of bivalents to multivalents as our estimator (Materials and Methods). This revealed a highly variable degree of stability in both *C. amara* cytotypes (mean proportion stable metaphase I cells in diploid maternal seed lines = 0.38–0.69, *n* = 133 scored cells; in tetraploids = 0.03–0.38; *n* = 348 scored cells; supplementary table 5, Supplementary Material online). Indeed, while still highly variable, the overall degree of stability was lower in autotetraploids versus diploids (differing proportion of stable to unstable meiotic cells for each ploidy; *D* = 62.7, df = 1, *P* < 0.0001, GLM with binomial errors; fig. 3*b*, supplementary table 5, Supplementary Material online), corresponding with the lack of selection signal in crossover-controlling meiosis genes. Interestingly, the broad variation in stability estimates within both cytotypes suggests widespread standing variation controlling this trait. In contrast, higher frequencies of stable metaphase I cells (>80%) have been commonly observed for diploid and autotetraploid *A. arenosa* (Marburger et al. 2019), although wider estimates of meiotic variation have also been observed in populations hybridizing with *A. lyrata* (Seear et al. 2020). Taken together with the observation of occasional clonal spreading of *C. amara* (Hejný et al. 1992; Tedder et al. 2015; Zozomová-Lihová et al. 2015), this indicates an ability to maintain stable populations, thus perhaps decreasing the immediate necessity to fully stabilize meiosis in either cytotype. Vegetative reproduction is often seen in polyploids (Herben et al. 2017; Van Drunen and Husband 2019) and in turn may have facilitated the establishment of the autotetraploid cytotypes. We note finally that the tetraploid populations are still highly fertile, consistent with observations across the range (Koch et al. 2003).

### Evidence for Process-level Convergence

Although we found no excess convergence at the level of orthologous genes under selection, we speculated that convergence may occur nevertheless at the level of functional processes. To test this, we used two complementary approaches: overlap of GO term enrichment and evidence of shared protein function from interaction networks. First, of 73 significantly (*P* < 0.05) enriched GO terms in *A. arenosa* (supplementary table 6, Supplementary Material online), we found that five were identical to those significantly enriched in *C. amara*, which is more than expected by chance (*P* < 0.001, Fisher’s exact test; table 3). In addition, some processes were found in both species, but were represented by slightly different terms, especially in the case of meiosis (“meiotic cell cycle” in *C. amara*, “meiotic cell cycle process” in *A. arenosa*: supplementary tables 3 and 6, Supplementary Material online). Remarkably, the relative ranking of enrichments of all five convergent terms was identical in both *C. amara* and *A. arenosa* (table 3). This stands in strong contrast to the fact that *A. arenosa* presented an obvious set of physically and functionally interacting genes in the top two categories (DNA metabolic process and chromosome organization), whereas the genes in these categories in *C. amara* are implicated in more diverse DNA management roles.

**Table 3.** Convergent Processes under Selection in Both *Cardamine amara* and *Arabidopsis arenosa* Following WGD.

GO ID	Term	*P*-value(*C. amara*)	*P*-value(*A. arenosa*)	Enrichment(*C. amara*)	Enrichment(*A. arenosa*)
GO:0006259	DNA metabolic process	6.50E-08	8.20E-04	3.72	2.46
GO:0051276	Chromosome organization	0.019	2.10E-04	1.98	2.01
GO:0009738	Abscisic acid-activated signaling pathway	0.032	0.022	2.54	2.10
GO:0071215	Cellular response to abscisic acid stimulation	0.048	0.04	2.30	1.90
GO:0097306	Cellular response to alcohol	0.048	0.04	2.30	1.90

Note.—*P*-values given are Fisher’s exact test, which tests for enrichment of terms from the GO hierarchy. Enrichment refers to fold enrichment.

**Fig. 4.**
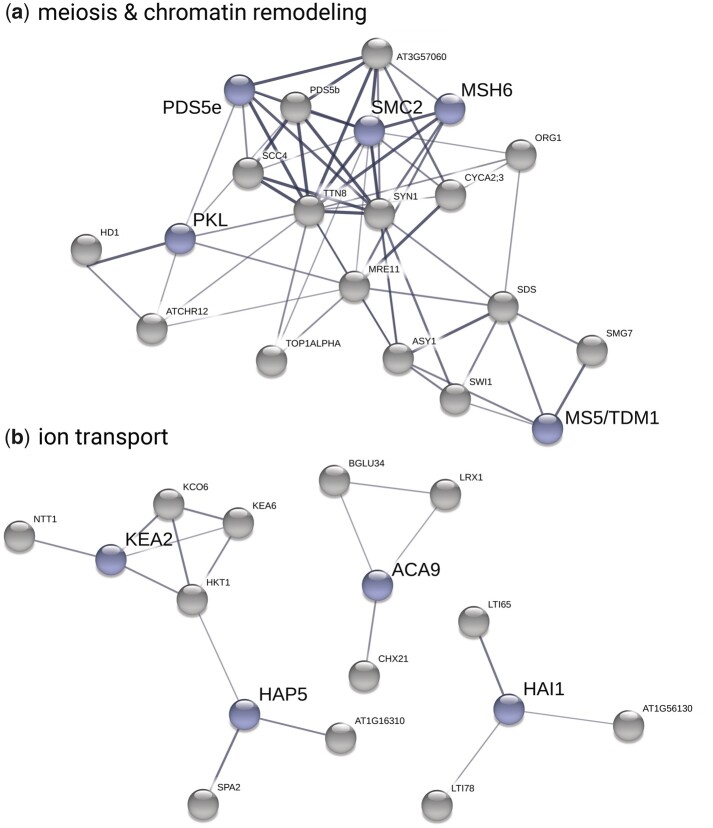
Evidence for functional convergence between *Cardamine amara* and *Arabidopsis arenosa* following independent WGDs. Plots show *C. amara* candidate genes in blue and STRING-associated *A. arenosa* candidate genes in gray. We used only medium confidence associations and higher (increasing thickness of lines connecting genes indicates greater confidence). (*a*) Meiosis- and chromatin remodeling-related genes. (*b*) Ion transport-related genes.

Second, we sought for evidence that genes under selection in *C. amara* might interact with those found under selection in *A. arenosa*, which would further support process-level convergence between the species. Thus, we took advantage of protein interaction information from the STRING database, which provides an estimate of proteins’ joint contributions to a shared function (Szklarczyk et al. 2015). For each *C. amara* WGD adaptation candidate we searched for the presence of STRING interactors among the *A. arenosa* WGD adaptation candidates, reasoning that finding such an association between candidates in two species may suggest that directional selection has targeted the same processes in both species through different genes. Following this approach, we found that out of the 229 *C. amara* WGD adaptation candidates, 90 were predicted to interact with at least one of the 452 WGD adaptation candidates in *A. arenosa*. In fact, 57 likely interacted with more than one *A. arenosa* candidate protein (fig. 4 and supplementary table 7, Supplementary Material online). This level of overlap was greater than expected by chance (*P* = 0.001 for both “any interaction” and “more-than-one interaction,” as determined by permutation tests with the same database and 1,000 randomly generated candidate lists).

Several large STRING clusters were evident among WGD adaptation candidates in *C. amara* and *A. arenosa* (fig. 4). The largest of these clusters center on genome maintenance, specifically meiosis and chromatin remodeling (fig. 4*a*), and ion homeostasis (especially K^+^ and Ca^2+^), along with stress (ABA) signaling (fig. 4*b*), consistent with the results of GO analysis. Taken together, both STRING and GO analyses support our hypothesis of functional convergence of these processes following WGD in *C. amara* and *A. arenosa.*

## Conclusions

Given the expected shared challenges attendant to WGD in *C. amara* and *A. arenosa*, we hypothesized at least partially convergent evolutionary responses to WGD. Although we found obvious convergent recruitment at the level of functional processes, we did not detect excess convergence at the gene level. This was consistent with the probable absence of shared standing variation between these species (Hudson and Coyne 2002), which are 17 My diverged. Nevertheless, we note that if any shared variation has persisted, it was not selected upon convergently in both young autotetraploids, thus strengthening the conclusion that the genes selected in response to WGD are not highly constrained.

The most prominent difference we observed here is the lack of an obvious coordinated evolutionary response in genes stabilizing early meiotic chromosome segregation in *C. amara*, relative to the striking coevolution of physically and functionally interacting proteins governing crossover formation in *A. arenosa*. This might be explained to some extent by our observation that in *C. amara* both diploids and autotetraploids are somewhat less meiotically stable than either cytotype in *A. arenosa*, and this instability may preadapt the autotetraploids to enjoy a less strict reliance on the generation of a high percentage of euploid gametes, by forcing occasional reliance on vegetative reproduction, as has been observed (Herben et al. 2017). This then may allow a decoupling of crossover number reduction from broader changes across meiosis and other processes we observe. This is not to say that we see no signal of WGD adaptation in *C. amara*: factors governing timing during later meiosis, especially the exit from meiotic divisions as evidenced by the interacting trio of *SMG7, SDS*, and *MS5*, along with other chromatin remodeling factors and DNA repair-related proteins, such as *MSH6* and *DAYSLEEPER* give very strong signals. The convergent functions we did detect (other meiotic processes, chromosome organization/chromatin remodeling, ABA signaling and ion transport) provide first insights into the salient challenges associated with WGD. We note also that tetraploid populations of both *C. amara* and *A. arenosa* are found in slightly colder environments than conspecific diploids (Zozomová-Lihová et al. 2015; Molina-Henao and Hopkins 2019), so some of these processes (e.g., ABA signaling and ion transport) might be linked to ecological adaptation following WGD.

Overall, our results provide contrast to widespread reports of gene-level convergence (reviewed, e.g., in Elmer and Meyer 2011; Martin and Orgogozo 2013; Blount et al. 2018) and support the idea that pathway-level convergence becomes dominant when the divergence between species is high (Takuno et al. 2015; Birkeland et al. 2020; Bohutínská et al. 2021b). This could be due to the absence of shared low-frequency alleles (acquired via gene flow or from standing variation) in species diverging millions of years ago, as was shown in alpine adaptation of different Brassicaceae species (Bohutínská et al. 2020). Alternatively, WGD provides complex multi-factorial challenge (Bomblies et al. 2015; Baduel, Bray, et al. 2018; Bomblies 2020) and the possible solutions to overcome such challenge may in fact be diverse. The result would be multiple alternative genetic paths to adaptation, with limited gene-level convergence due to the low diversity constraints (Yeaman et al. 2018). Finally, we note that the lack of gene-level convergence in meiosis genes suggests that the genomic changes associated with meiosis stabilization after WGD might not be as constrained as would be expected based on its functional conservation across eukaryotes (Grishaeva and Bogdanov 2014; Rosenberg and Corbett 2015; Baker et al. 2017).

We conclude that evolutionary solutions to WGD-associated challenges vary strongly from case to case, suggesting less functional constraint than one may expect based on the fact that these processes are conserved and essential. This may help explain how it is that many species manage to thrive following WGD and, once established as polyploids, experience evolutionary success. In fact, we envision that the meiotic instability experienced by some WGD lineages, such as *C. amara*, could serve as a diversity-generating engine promoting large effect genomic structural variation, as has been observed in aggressive polyploid gliomas (Yant and Bomblies 2015).

## Materials and Methods

### Reference Genome Assembly and Alignment

We generated a de novo assembly using the 10× Genomics Chromium approach. In brief, a single diploid individual from pop LUZ (supplementary table 8, Supplementary Material online) was used to generate one Chromium library, sequenced using 250PE mode on an Illumina sequencer, and assembled with Supernova version 2.0.0. This assembly had an overall scaffold N50 of 1.82 Mb. An assessment of genome completeness using BUSCO (version 3.0.2) (Seppey et al. 2019) for the 2,251 contigs ≥ 10 kb was estimated at 94.8% (1,365/1,440 BUSCO groups; supplementary table 9, Supplementary Material online). 

### BioNano Plant Extraction Protocol

Fresh young leaves of the *C. amara* accession LUZ were collected after 48-h dark treatment. DNA was extracted by the Earlham Institute’s Platforms and Pipelines group following an IrysPrep “FixnBlend” Plant DNA extraction protocol supplied by BioNano Genomics. First 2.5 g of fresh young leaves were fixed with 2% formaldehyde. After washing, leaves were disrupted and homogenized in the presence of an isolation buffer containing PVP10 and BME to prevent polyphenol oxidation. Triton X-100 was added to facilitate nuclei release. Nuclei were then purified on a Percoll cushion. The nuclear phase was taken and washed in isolation buffer before embedding into low melting point agarose. Two plugs of 90 μl were cast using the CHEF Mammalian Genomic DNA Plug Kit (Bio-Rad 170-3591). Once set at 4 °C the plugs were added to a lysis solution containing 200 μl proteinase K (QIAGEN 158920) and 2.5 ml of BioNano lysis buffer in a 50 ml conical tube. These were put at 50 °C for 2 h on a thermomixer, making a fresh proteinase K solution to incubate overnight. Samples were then removed from the thermomixer for 5 min before 50 μl RNAse A (Qiagen158924) was added and the tubes incubated for a further hour at 37 °C. Plugs were then washed 7 times in the Wash Buffer supplied in the Chef kit and 7 times in 1×TE. One plug was removed and melted for 2 min at 70 °C followed by 5 min at 43 °C before adding 10 μl of 0.2 U/μl of GELase (Cambio Ltd G31200). After 45 min at 43 °C the melted plug was dialyzed on a 0.1 μM membrane (Millipore VCWP04700) sitting on 15 ml of 1×TE in a small petri dish. After 2 h the sample was removed with a wide bore tip and mixed gently and left overnight at 4 °C.

### 10× Library Construction

DNA material was diluted to 0.5 ng/µl with EB (Qiagen) and checked with a QuBit Flourometer 2.0 (Invitrogen) using the QuBit dsDNA HS Assay kit (supplementary table 8, Supplementary Material online). The Chromium User Guide was followed as per the manufacturer’s instructions (10× Genomics, CG00043, Rev A). The final library was quantified using qPCR (KAPA Library Quant kit [Illumina] and ABI Prism qPCR Mix, Kapa Biosystems). Sizing of the library fragments was checked using a Bioanalyzer (High Sensitivity DNA Reagents, Agilent). Samples were pooled based on the molarities calculated using the two QC measurements. The library was clustered at 8 pM with a 1% spike in of PhiX library (Illumina). The pool was run on a HiSeq2500 250 bp Rapid Run V2 mode (Illumina).

### Sequencing and Assembly

Reads were subsampled to 90 M reads and assembled with Supernova 2.0.0 (10× Genomics), giving a raw coverage of 60.30× and an effective coverage of 47.43×. The estimated molecule length was 44.15 kb. The assembly size, considering only scaffolds longer than 10 kb was 159.53 Mb and the Scaffold N50 was 1.82 Mb. Genome size estimate by kmer analysis was 225.39 Mb, hence we estimate we are missing 16.61% from the assembly. Because the diploid individual used for reference genome sequencing was not homozygous, we sought to confirm whether the assembly harbored evidence of uncollapsed haplotypes by using a reciprocal BLAST (Camacho et al. 2009) best hits approach. A small proportion (1.7%) of scaffolds exhibited substantial homology (90% or greater identity to another scaffold over 90% of their length), indicating that very few alternate alleles at heterozygous loci were misinferred as separate genomic loci in the diploid assembly. Manual investigation of a suite of meiosis-related loci indicated no cases of false negatives in the data set caused by alternate alleles aligning to separate scaffolds. We further scaffolded the assembly using the published *Cardamine hirsuta* genome using *graphAlign* (Spalding and Lammers 2004) and *Nucmer* (Marçais et al. 2018).

### Gene Calling and Annotation

The plants set database embryophyta_odb9.tar.gz was downloaded from http://busco.ezlab.org/ and used to assess orthologue presence/absence in our *C. amara* genome annotation. Running BUSCO gave Augustus (Stanke and Waack 2003) results via BUSCO HMMs to infer where genes lie in the assembly and to infer protein sequences. Augustus was used to generate a gff annotation file using “arabidopsis” as the training option. A BLAST (v. 2.2.4) database was built for Brassicales (taxid: 3699) by downloading approximately 1.26 M protein sequences from https://www.ncbi.nlm.nih.gov/taxonomy/ and the Augustus-predicted proteins were annotated via Interproscan (Quevillon et al. 2005) and blast2go (Conesa and Götz 2008).

### Functional Annotation of *C. amara* Genes

To functionally annotate *C. amara* genes we performed an orthogrouping analysis using Orthofinder version 2.3.3 (Emms and Kelly 2018), inferring orthologous groups (OGs) from four species (*C. amara, A. lyrata, A. thaliana, Cochlearia pyrenaica*). A total of 21,618 OGs were found. Best reciprocal BLAST hits (RBHs) for *C. amara* and *A. thaliana* genes were found using BLAST version 2.9.0.

*Cardamine amara* genes were then assigned an *A. thaliana* gene ID for GO enrichment analysis via the following protocol: 1) if the *C. amara* gene was in an OG with only one *A. thaliana* gene, that *A. thaliana* ID was used; 2) if the *C. amara* gene was in an OG with more than one *A. thaliana* gene, then the RBH, provided it was in the same OG with the *C. amara* gene, was used; 3) if the *C. amara* gene was in an OG that contained more than one *A. thaliana* gene, none of which was the RBH, then the *A. thaliana* gene from that OG with the lowest BLAST E-value was taken; 4) if the *C. amara* gene was in an OG group that lacked *A. thaliana* genes, then the RBH was taken instead; and 5) finally, if the *C. amara* gene was in an OG group without any *A. thaliana* genes and there was no RBH, then the gene with the lowest E-value in a BLASTs versus the TAIR10 database was used. BLASTs versus the TAIR10 database were performed during December 2019.

### Sampling, Sequencing, and Genetic Structure Analysis

#### Sampling

A total of 100 plants were sampled from four populations (fig. 1*d*): 25 individuals for each of CEZ (4×), PIC (4×), VKR (2×), and LUZ (2×). Sampled plants were spaced at least 3 m apart, as such distance was enough to avoid resampling of identical clones according to analysis in a study sampling approximately 3,300 individuals across the *C. amara* range, including these populations (Zozomová-Lihová et al. 2015).

#### Flow Cytometry

All plants used for DNA extraction were verified for expected ploidy by flow cytometry. Approximately 1 square cm of leaf material was diced alongside an internal reference using a razor blade in 1 ml ice cold extraction buffer (45 mM MgCl_2_, 30 mM sodium citrate, 20 mM MOPS, 1% Triton-100, pH 7 with NaOH). The resultant slurry was then filtered through a 40-μm nylon mesh before the nuclei were stained with the addition of 1 ml staining buffer (either CyStain UV precise P [Sysmex, Fluorescence emission: 435–500 nm] for relative ploidy, or Otto 2 buffer [0.4 M Na_2_HPO_4_·12H_2_O, Propidium iodide 50 μg/ml, RNase 50 μg/ml], for absolute DNA content). After 1 min of incubation at room temperature the sample was run for 5,000 particles on either a Partec PA II flow cytometer or a BD FACS Melody. Histograms were evaluated using FlowJo software version 10.6.1.

#### DNA Isolation, Library Preparation, and Sequencing

A replicated approach was used for the DNA isolation, pooling, and sequencing to reduce variation that may be associated with Pool-Seq data. DNA isolations were performed in triplicate for every plant and then each replicate was pooled with samples from the other 24 replicates in each population, generating three independently extracted and pooled replicates for every population. DNA was extracted with the RNeasy Plant Mini Kit (Qiagen). Each of the 12 resultant pools for the four populations was used as input for library construction with the Illumina Truseq kit (Illumina, Inc.), and then sequenced on an Illumina NextSeq (150 bp paired end specification).

#### Data Preparation, Alignment, and Genotyping

Fastq files from two runs on the Illumina NextSeq concatenated to give an average of 30.5 million reads per sample. Adapter sequences were removed using cutadapt (version 1.9.1) (Martin 2011) and quality trimmed via Sickle (version 33) (Joshi and Fass 2011) to generate only high-quality reads (Phred score ≥30) of 30 bp or more, resulting in an average of 27.9 million reads per sample. Reads were then aligned with (Li et al. 2009) BWA (version 0.7.12) (Li and Durbin 2009) and processed with Samtools (version 1.7) (Li et al. 2009). Using Picard (version 1.134) (Broad Institute 2009), duplicate reads were removed via MarkDuplicates followed by the addition of read group IDs to the bam files via AddOrReplaceReadGroups. Finally, to handle the presence of indels, GATK (version 3.6.0) (McKenna et al. 2010) was used to realign reads using IndelRealigner.

#### Variant Calling

Variants were called for the 12 bam files (three replicates per population) using Freebayes (version 1.1.0.46) (Garrison and Marth 2012) to generate a single VCF output file. Freebayes was run with default parameters, except we specified “--pooled-discrete” to indicate samples were pooled, “--use-best-n-alleles 2” to restrict to biallelic sites, and “--no-indels” to exclude indels. The resultant VCF was then filtered with BCFtools (version 1.8) (Narasimhan et al. 2016) to remove sites where the read depth was <10, or >1.6× the second mode (determined as 1.6 × 31 = 50, supplementary fig. 3, Supplementary Material online) in order to remove from the analysis regions exhibiting heterozygous deletions or where multiple genomic regions may have mapped to the reference due to, for example, paralogous duplications n the sequenced individuals.

#### Population Genetic Structure

We first calculated genome-wide between-population metrics (Nei’s *F*_st_ [Nei 1972] and AF difference). The AF in individual replicate pools was calculated as the fraction of the total number of reads supporting the alternative allele (Anand et al. 2016). For each population the average AF was then calculated from the three replicates and used for all further calculations. We used the python3 PoolSeqBPM pipeline, designed to input pooled data (https://github.com/mbohutinska/PoolSeqBPM). Then we inferred relationships between populations over putatively neutral 4-fold degenerate SNPs using PCA as implemented in *adegenet* (Jombart and Ahmed 2011). Finally, we inferred relationships between populations using AF covariance graphs implemented in TreeMix (Pickrell and Pritchard 2012). We ran TreeMix allowing a range of migration events; and presented one additional migration edge, as it represented points of log-likelihood saturation. To obtain confidence in the reconstructed topology, we bootstrapped the scenario with zero events (the tree topology had not changed when considering the migration events) choosing a bootstrap block size of 1,000 bp, equivalent to the window size in our selection scan, and 100 replicates.

### Genome Scans for Selection

To detect signals of selection, we used a combination of two different selection scan approaches. First, we calculated pairwise window-based *F*_st_ between diploid and populations and used minimum sum of ranks between informative contrasts in a quartet design (below). To further control for false positives and refine the gene list to putatively functional candidates we complemented these differentiation measures with a functional score estimate following the FineMAV method (below). Both approaches are based on population AFs and allow analysis of diploid and autopolyploid populations.

#### Window-Based Selection Scan Using a Quartet Design

We performed a window-based *F*_st_ (Nei 1972) scan for directional selection in *C. amara*, taking advantage of quartet sampling of two diploid and two autotetraploid populations (fig. 1*d*). Using this design, we identified top candidate windows for selective sweeps associated with ploidy differentiation, while excluding differentiation patterns private to a single population or ploidy-uninformative selective sweeps. Thus comparisons between populations of the same ploidy constitute a null model for shared heterogeneity in genetic differentiation arising through processes unrelated to WGD (following an approach successfully applied in Vijay et al. 2016). To do this, we calculated *F*_st_ for 1-kb windows with minimum 20 SNPs for all six population pairs in the quartet (fig. 1*d*) and ranked windows based on their *F*_st_ value. We excluded windows which were top 1% outliers in diploid–diploid (dd in fig. 1*d*) or autotetraploid–autotetraploid (tt) population contrasts, as they represent variation inconsistent with diploid–autotetraploid divergence but rather signal local differentiation within a cytotype. Next, we assigned ranks to each window based on the *F*_st_ values in four diploid–autotetraploid contrasts and identified windows being top 1% outliers of minimum rank sum.

Because candidate detection could be biased by arbitrary window size choice, we re-analyzed our differentiation scans changing two parameters: 1) using a SNP-by-SNP basis (requiring at least five SNPs per gene for inclusion); and 2) using larger, 50-kb windows. Doing this, we found that SNP-level and 1-kb-window scans resulted in comparable candidate gene lists, whereas 50-kb windows were too wide to identify local peaks of differentiation. Thus, we decided to use scans with a window size of 1 kb, which best corresponded to the average length of selective sweep signatures in differentiation plots (e.g., fig. 2), and allowed to locate the candidate selected region while still providing enough polymorphisms to robustly estimate differentiation between ploidies.

To account for possible confounding effect of comparing windows from genic and nongenic regions, we calculated the number of base pairs overlapping with any gene within each window. There was no relationship between the proportion of genic space within a window and *F*_st_ (Pearsons *r* = −0.057, supplementary fig. 4, Supplementary Material online), indicating that our analyses were unaffected by unequal proportion of genic space in a window.

In *A. arenosa*, we performed window-based *F*_st_ scan for directional selection using the same criteria as for *C. amara* (1-kb windows, min 20 SNPs per window). We did not use the quartet design as the range-wide data set of 80 diploid and 40 autotetraploid individuals drawn from the entire *A. arenosa* range (15 diploid and 24 autotetraploid populations) assured power to detect genomic regions with WGD-associated differentiation. This *A. arenosa* analysis gave very similar results to (Yant et al. 2013), which used only two diploid and four autotetraploid populations, indicating minimal dependence on sampling to detect these strongest signatures of selection in the *A. arenosa* system.

#### FineMAV

We adopted the approach, Fine-Mapping of Adaptive Variation, FineMAV (Szpak et al. 2018), using our *C. amara* annotation (following approach successfully applied to nonhuman genome in Bohutínská et al. 2021a). To functionally annotate each amino acid change, we used the Grantham score (Grantham 1974), a theoretical amino acid substitution value, encoded in the Grantham matrix, where each element shows the differences of physicochemical properties between two amino acids. We used SnpEff (version 4.3) (Cingolani et al. 2012) to annotate our SNP data set by applying our Augustus-generated *C. amara* annotation (“Gene Calling and Annotation,” above). We estimated the population genetic component of FineMAV (see [Szpak et al. 2018] for details on calculations) using AF information at each site (considering minor frequency alleles as derived) and derived allele purity (DAP) parameter of 3.5, a measure of population differentiation, which describes how unequally the derived allele is distributed among populations. The advantage is that DAP can summarize differentiation across many populations in a single measure for each variant. Finally, for each amino acid substitution, we assigned Grantham scores, together with population genetic component of FineMAV, using custom scripts in Python 2.7.10 and Biopython version 1.69. We identified the top 1% outliers as FineMAV candidates. All calculations were performed using code available at (github.com/paajanen/meiosis_protein_evolution).

#### *Arabidopsis arenosa* Population Genomic Data Set

Our selection analysis in *A. arenosa* was based on an expanded sampling (Monnahan et al. 2019) relative to Yant et al. (2013), who sampled 24 individuals (from two diploid and four tetraploid populations, sourced from a fraction of now known lineages). This expanded sampling covered all known lineages, across the entire range of the species, including 39 populations: 15 diploid populations (105 individually resequenced plants) and 24 tetraploid populations (182 individually resequenced plants) (Monnahan et al. 2019). We aligned PE Illumina data to the *A. lyrata* reference (Hu et al. 2011), called variants and filtered as previously (Monnahan et al. 2019) using GATK 3.5 (McKenna et al. 2010). We used a subset of the data consisting of 80 diploid individuals and 40 tetraploid individuals from populations unaffected by secondary introgression from diploid lineages (following Bohutínská et al. 2021a; samples selected based on the highest mean depth of coverage). Such subsampling gave us a balanced number of 160 high-quality haploid genomes of each ploidy suitable for selection scans. Finally, we filtered each subsampled data set for genotype read depth >8 and maximum fraction of missing genotypes < 0.5 in each lineage. We calculated *F*_st_ using python3 ScanTools pipeline (github.com/mbohutinska/ScanTools_ProtEvol). All subsequent analyses were performed following the same procedure as with *C. amara* data.

#### GO Enrichment Analysis

To infer functions significantly associated with directional selection following WGD, we performed a GO enrichment on the gene list using the R package topGO (Tilford and Siemers 2009), using *A. thaliana* orthologs of *C. amara/A. lyrata* genes, obtained using biomaRt (Smedley et al. 2009). We used Fisher’s exact test with conservative “elim” method, which tests for enrichment of terms from the bottom of the GO hierarchy to the top and discards any genes that are significantly enriched in a descendant GO terms (Grossmann et al. 2007). Re-analysis with the “classic” method did not identify any additional convergently enriched GO terms. We used biological process ontology with minimum node size of 150 genes.

#### Protein Associations from STRING Database

We searched for potential functional associations among *C. amara* and *A. arenosa* candidate genes using STRING (Szklarczyk et al. 2015). Genes were assigned an *A. thaliana* gene ID as described above. We used the “multiple proteins” search in *A. thaliana*, with text mining, experiments, databases, co-expression, neighborhood, gene fusion and co-occurrence as information sources. We used minimum confidence 0.4 and retained only 1st shell associations (proteins that are directly associated with the candidate protein: i.e., immediately neighboring network circles).

#### Quantifying Convergence

We considered convergent any candidates or enriched GO categories that overlapped across both species. Convergent candidate genes had to be members of the same orthogroups (Emms and Kelly 2018). To test for higher than random number of overlapping items we used Fisher’s Exact Test for Count Data in R (R Development Core Team 2011).

### Cytological Assessment of Meiotic Stability

We cytologically estimated the degree of male meiotic stability in *C. amara* by counting the number of bivalent chromosome associations in each metaphase event. A lower number of bivalents and a higher number of multivalents are taken as a proxy for reduced meiotic stability. The reasoning behind this is that a reduction in crossover number to one per bivalent is strongly indicated as a leading mechanism for meiotic diploidization in autopolyploids as this limits multivalent associations (which increase the propensity toward breakage and aneuploidy vs. bivalents [Cifuentes et al. 2010; Le Comber et al. 2010; Bomblies et al. 2016]).

#### Chromosome Preparation

Whole young inflorescences were fixed in freshly prepared ethanol: acetic acid (3:1) overnight, transferred into 70% ethanol and stored at –20 °C until use. Meiotic chromosome spreads were prepared from anthers according to Mandáková et al. (2014). Briefly, after washing in citrate buffer (10 mM sodium citrate, pH 4.8), selected flower buds were digested using a 0.3% mix of pectolytic enzymes (cellulase, cytohelisase, pectolyase; Sigma–Aldrich Corp., St. Louis, MO) in citrate buffer for 3 h. Individual anthers were dissected and spread in 20 µl of 60% acetic acid on a microscope slide placed on a metal hot plate (50 °C), fixed by ethanol: acetic acid (3:1) and the preparation was dried using a hair dryer. Slides were postfixed in freshly prepared 4% formaldehyde in distilled water for 10 min and air-dried. The preparations were stained with 4′,6-diamidino-2-phenylindole (DAPI; 2 µg/ml) in Vectashield (Vector Laboratories, Peterborough, UK). Fluorescence signals were analyzed using an Axioimager Z2 epifluorescence microscope (Zeiss, Oberkochen, Germany) and CoolCube CCD camera (MetaSystems, Newton, MA).

#### Meiotic Stability Assessments

In diploids, chromosome spreads with 8 bivalents were scored as “stable meiosis,” 7-6 as “partly stable,” 5-4 as “partly unstable,” and <4 as “unstable.” In autotetraploids, chromosome spreads with 16 bivalents were scored as “stable meiosis,” 14-12 as “partly stable,” 10-8 as “partly unstable,” and <8 as “unstable.” We report a mean value of meiotic stability for each ploidy calculated over “stable meiosis” and over sum of “stable meiosis” and “partly stable” categories. Differences in meiotic stability between diploids and autotetraploids (fig. 3*b*) are reported for the sum of “stable” and “partly stable” categories. However, considering only the “stable meiosis” category does not qualitatively affect the results (i.e., the degree of meiotic stability is significantly lower in tetraploids, *D* = 125.7, df = 1, *P* < 0.0001, GLM with binomial errors). Photos of all spreads scored are supplied in supplementary figure 4, Supplementary Material online. 

## Supplementary Material

Supplementary data are available at *Molecular Biology and Evolution* online. 

## Supplementary Material

msab096_Supplementary_Data

## References

[msab096-B1] Aichinger E, Villar CBR, di Mambro R, Sabatini S, Köhler C. 2011. The CHD3 chromatin remodeler PICKLE and polycomb group proteins antagonistically regulate meristem activity in the Arabidopsis root. Plant Cell. 23(3):1047–1060. 21441433 10.1105/tpc.111.083352 PMC3082253

[msab096-B2] Anand S, Mangano E, Barizzone N, Bordoni R, Sorosina M, Clarelli F, Corrado L, Boneschi FM, D’Alfonso S, De BG. 2016. Next generation sequencing of pooled samples: guideline for variants’ filtering. Sci Rep. 6:33735. 10.1038/srep33735 PMC5037392 27670852

[msab096-B3] Arnold B, Kim ST, Bomblies K. 2015. Single geographic origin of a widespread autotetraploid *Arabidopsis arenosa* lineage followed by interploidy admixture. Mol Biol Evol. 32(6):1382–1395. 25862142 10.1093/molbev/msv089

[msab096-B4] Arnold BJ, Lahner B, DaCosta JM, Weisman CM, Hollister JD, Salt DE, Bomblies K, Yant L. 2016. Borrowed alleles and convergence in serpentine adaptation. Proc Natl Acad Sci USA. 113(29):8320–8325. 27357660 10.1073/pnas.1600405113 PMC4961121

[msab096-B5] Baduel P, Bray S, Vallejo-Marin M, Kolář F, Yant L. 2018. The “Polyploid Hop”: shifting challenges and opportunities over the evolutionary lifespan of genome duplications. Front Ecol Evol. 6:117.

[msab096-B6] Baduel P, Hunter B, Yeola S, Bomblies K. 2018. Genetic basis and evolution of rapid cycling in railway populations of tetraploid *Arabidopsis arenosa*. PLoS Genet. 14(7):e1007510. 29975688 10.1371/journal.pgen.1007510 PMC6049958

[msab096-B7] Baker Z, Schumer M, Haba Y, Bashkirova L, Holland C, Rosenthal GG, Przeworski M. 2017. Repeated losses of PRDM9-directed recombination despite the conservation of PRDM9 across vertebrates. Elife 6:e24133. 10.7554/eLife.24133 PMC5519329 28590247

[msab096-B8] Birkeland S, Gustafsson ALS, Brysting AK, Brochmann C, Nowak MD, Purugganan M. 2020. Multiple genetic trajectories to extreme abiotic stress adaptation in arctic Brassicaceae. Mol Biol Evol. 37(7):2052–2068. 32167553 10.1093/molbev/msaa068 PMC7306683

[msab096-B9] Camacho C, Coulouris G, Avagyan V, Ma N, Papadopoulos J, Bealer K, Madden TL. 2009. BLAST+: architecture and applications. *BMC Bioinformatics*. 10:421. 10.1186/1471-2105-10-421 PMC2803857 20003500

[msab096-B10] Blount ZD, Lenski RE, Losos JB. 2018. Contingency and determinism in evolution: replaying life’s tape. Science 362(6415):eaam5979. 30409860 10.1126/science.aam5979

[msab096-B11] Bohutínská M, Handrick V, Yant L, Schmickl R, Kolář F, Bomblies K, Paajanen P. 2021a. De-novo mutation and rapid protein (co-)evolution during meiotic adaptation in *Arabidopsis arenosa*. Mol Biol Evol. 38(5):1980–1994. 33502506 10.1093/molbev/msab001 PMC8097281

[msab096-B12] Bohutínská M, Vlček J, Yair S, Laenen B, Konečná V, Fracassetti M, Slotte T, Kolář F. 2021b. Genomic basis of parallel adaptation varies with divergence in *Arabidopsis* and its relatives. *Proc Natl Acad Sci U S A*. 118(21)e2022713118. 10.1073/pnas.2022713118 PMC8166048 34001609

[msab096-B13] Bomblies K. 2020. When everything changes at once: finding a new normal after genome duplication: evolutionary response to polyploidy. Proc R Soc B Biol Sci. 287: 20202154. 10.1098/rspb.2020.2154 PMC7739491 33203329

[msab096-B14] Bomblies K, Higgins JD, Yant L. 2015. Meiosis evolves: adaptation to external and internal environments. New Phytol. 208(2):306–323. 26075313 10.1111/nph.13499

[msab096-B15] Bomblies K, Jones G, Franklin C, Zickler D, Kleckner N. 2016. The challenge of evolving stable polyploidy: could an increase in “crossover interference distance” play a central role? Chromosoma 125(2):287–300. 26753761 10.1007/s00412-015-0571-4 PMC4830878

[msab096-B16] Bomblies K, Madlung A. 2014. Polyploidy in the Arabidopsis genus. Chromosome Res. 22(2):117–134. 24788061 10.1007/s10577-014-9416-x

[msab096-B17] Broad Institute 2009. “Picard Tools.” Broad Institute, GitHub repository. Available from: http://broadinstitute.github.io/picard/.

[msab096-B18] Bundock P, Hooykaas P. 2005. An Arabidopsis hAT-like transposase is essential for plant development. Nature 436(7048):282–284. 16015335 10.1038/nature03667

[msab096-B19] Chao DY, Dilkes B, Luo H, Douglas A, Yakubova E, Lahner B, Salt DE. 2013. Polyploids exhibit higher potassium uptake and salinity tolerance in Arabidopsis. Science 341(6146):658–659. 23887874 10.1126/science.1240561 PMC4018534

[msab096-B20] Cifuentes M, Grandont L, Moore G, Chèvre AM, Jenczewski E. 2010. Genetic regulation of meiosis in polyploid species: new insights into an old question. New Phytol. 186(1):29–36. 19912546 10.1111/j.1469-8137.2009.03084.x

[msab096-B21] Cifuentes M, Jolivet S, Cromer L, Harashima H, Bulankova P, Renne C, Crismani W, Nomura Y, Nakagami H, Sugimoto K, et al 2016. TDM1 regulation determines the number of meiotic divisions. PLoS Genet. 12(2):e1005856. 26871453 10.1371/journal.pgen.1005856 PMC4752240

[msab096-B22] Cingolani P, Platts A, Wang LL, Coon M, Nguyen T, Wang L, Land SJ, Lu X, Ruden DM. 2012. A program for annotating and predicting the effects of single nucleotide polymorphisms, SnpEff: SNPs in the genome of Drosophila melanogaster strain w1118; iso-2; iso-3. Fly (Austin). 6(2):80–92. 22728672 10.4161/fly.19695 PMC3679285

[msab096-B23] Colmenero-Flores JM, Martínez G, Gamba G, Vázquez N, Iglesias DJ, Brumós J, Talón M. 2007. Identification and functional characterization of cation-chloride cotransporters in plants. Plant J. 50(2):278–292. 17355435 10.1111/j.1365-313X.2007.03048.x

[msab096-B24] Conesa A, Götz S. 2008. Blast2GO: a comprehensive suite for functional analysis in plant genomics. Int J Plant Genomics. 2008:619832. 18483572 10.1155/2008/619832 PMC2375974

[msab096-B25] Culligan KM, Hays JB. 2000. Arabidopsis MutS homologs – AtMSH2, AtMSH3, AtMSH6, and a novel AtMSH7 – form three distinct protein heterodimers with different specificities for mismatched DNA. Plant Cell. 12:991–1002. 10852942 10.1105/tpc.12.6.991 PMC149098

[msab096-B26] Cutler SR, Rodriguez PL, Finkelstein RR, Abrams SR. 2010. Abscisic acid: emergence of a core signaling network. Annu Rev Plant Biol. 61:651–679. 20192755 10.1146/annurev-arplant-042809-112122

[msab096-B27] Doyle JJ, Coate JE. 2019. Polyploidy, the nucleotype, and novelty: the impact of genome doubling on the biology of the cell. Int J Plant Sci. 180(1):1–52.

[msab096-B28] Elmer KR, Meyer A. 2011. Adaptation in the age of ecological genomics: insights from parallelism and convergence. Trends Ecol Evol. 26(6):298–306. 21459472 10.1016/j.tree.2011.02.008

[msab096-B29] Emms DM, Kelly S. 2018. OrthoFinder: Phylogenetic orthology inference for comparative genomics. *bioRxiv* 466201. Available from: https://www.biorxiv.org/content/10.1101/466201v1. 10.1186/s13059-019-1832-y PMC6857279 31727128

[msab096-B30] Fujii H, Verslues PE, Zhu JK. 2011. Arabidopsis decuple mutant reveals the importance of SnRK2 kinases in osmotic stress responses in vivo. Proc Natl Acad Sci USA. 108(4):1717–1722. 21220313 10.1073/pnas.1018367108 PMC3029766

[msab096-B32] Garrison E, Marth G. 2012. Haplotype-based variant detection from short-read sequencing. Cornell University. Available from: http://arxiv.org/abs/1207.3907.

[msab096-B33] Glover J, Grelon M, Craig S, Chaudhury A, Dennis E. 1998. Cloning and characterization of MS5 from Arabidopsis: a gene critical in male meiosis. Plant J. 15(3):345–356. 9750346 10.1046/j.1365-313x.1998.00216.x

[msab096-B34] Grantham R. 1974. Amino acid difference formula to help explain protein evolution. Science 185(4154):862–864. 4843792 10.1126/science.185.4154.862

[msab096-B35] Grishaeva TM, Bogdanov YF. 2014. Conservation and variability of synaptonemal complex proteins in phylogenesis of Eukaryotes. Int J Evol Biol. 2014:856230–856216. 25147749 10.1155/2014/856230 PMC4132317

[msab096-B36] Grossmann S, Bauer S, Robinson PN, Vingron M. 2007. Improved detection of overrepresentation of Gene-Ontology annotations with parent-child analysis. Bioinformatics 23(22):3024–3031. 17848398 10.1093/bioinformatics/btm440

[msab096-B37] Hejný S, Slavík B, Kirschner J, Křísa B. 1992. Květena České republiky 3. Prague, Czech Republic: Academia.

[msab096-B38] Herben T, Suda J, Klimešová J. 2017. Polyploid species rely on vegetative reproduction more than diploids: a re-examination of the old hypothesis. Ann Bot. 120(2):341–349. 28334206 10.1093/aob/mcx009 PMC5737615

[msab096-B39] Hollister JD, Arnold BJ, Svedin E, Xue KS, Dilkes BP, Bomblies K. 2012. Genetic adaptation associated with genome-doubling in autotetraploid *Arabidopsis arenosa*. PLoS Genet. 8(12):e1003093. 23284289 10.1371/journal.pgen.1003093 PMC3527224

[msab096-B40] Hu TT, Pattyn P, Bakker EG, Cao J, Cheng JF, Clark RM, Fahlgren N, Fawcett JA, Grimwood J, Gundlach H, et al 2011. The *Arabidopsis lyrata* genome sequence and the basis of rapid genome size change. Nat Genet. 43(5):476–483. 21478890 10.1038/ng.807 PMC3083492

[msab096-B41] Huang XC, German DA, Koch MA. 2020. Temporal patterns of diversification in Brassicaceae demonstrate decoupling of rate shifts and mesopolyploidization events. Ann Bot. 125(1):29–47. 31314080 10.1093/aob/mcz123 PMC6948214

[msab096-B42] Hudson RR, Coyne JA. 2002. Mathematical consequences of the genealogical species concept. Evolution (NY) 56:1557–1565. 10.1111/j.0014-3820.2002.tb01467.x 12353748

[msab096-B43] Jing Y, Guo Q, Lin R. 2019. The chromatin-remodeling factor pickle antagonizes polycomb repression of FT to promote flowering. Plant Physiol. 181(2):656–668. 31377725 10.1104/pp.19.00596 PMC6776858

[msab096-B44] Jombart T, Ahmed I. 2011. adegenet 1.3-1: new tools for the analysis of genome-wide SNP data. Bioinformatics 27(21):3070–3071. 21926124 10.1093/bioinformatics/btr521 PMC3198581

[msab096-B45] Joshi N, Fass J. 2011. Sickle: A sliding-window, adaptive, quality-based trimming tool for FastQ files (Version 1.33) [Software]. Available from: https://github.com/najoshi/sickle.:2011.

[msab096-B46] Kang X, Xu G, Lee B, Chen C, Zhang H, Kuang R, Ni M. 2018. HRB2 and BBX21 interaction modulates Arabidopsis ABI5 locus and stomatal aperture. Plant Cell Environ. 41(8):1912–1925. 29748960 10.1111/pce.13336

[msab096-B47] Kawa D, Meyer AJ, Dekker HL, Abd-El-Haliem AM, Gevaert K, Van De Slijke E, Maszkowska J, Bucholc M, Dobrowolska G, De Jaeger G, et al 2020. SnRK2 protein kinases and mRNA decapping machinery control root development and response to salt. Plant Physiol. 182(1):361–371. 31570508 10.1104/pp.19.00818 PMC6945840

[msab096-B48] Klepikova AV, Kasianov AS, Gerasimov ES, Logacheva MD, Penin AA. 2016. A high resolution map of the *Arabidopsis thaliana* developmental transcriptome based on RNA-seq profiling. Plant J. 88(6):1058–1070. 27549386 10.1111/tpj.13312

[msab096-B49] Knip M. (Leiden U). 2012. Daysleeper: from genomic parasite to indispensable gene. Leiden: LEI Universiteit. Available from: https://hdl.handle.net/1887/20170

[msab096-B50] Koch M, Huthmann M, Bernhardt KG. 2003. *Cardamine amara* L. (Brassicaceae) in dynamic habitats: genetic composition and diversity of seed bank and established populations. Basic Appl Ecol. 4(4):339–348.

[msab096-B51] Kolář F, Fuxová G, Záveská E, Nagano AJ, Hyklová L, Lučanová M, Kudoh H, Marhold K. 2016. Northern glacial refugia and altitudinal niche divergence shape genome-wide differentiation in the emerging plant model *Arabidopsis arenosa*. Mol Ecol. 25(16):3929–3949. 27288974 10.1111/mec.13721

[msab096-B52] Konečná V, Bray S, Vlček J, Bohutínská M, Požárová D, Choudhury RR, Bollmann-Giolai A, Flis P, Salt DE Parisod C, et al 2021. Parallel adaptation in autopolyploid *Arabidopsis arenosa* is dominated by repeated recruitment of shared alleles. *bioRxiv* [Internet]:2021.01.15.426785. Available from: http://biorxiv.org/content/early/2021/01/17/2021.01.15.426785.abstract. 10.1038/s41467-021-25256-5 PMC8370997 34404804

[msab096-B53] Kunz HH, Gierth M, Herdean A, Satoh-Cruz M, Kramer DM, Spetea C, Schroeder JI. 2014. Plastidial transporters KEA1, -2, and -3 are essential for chloroplast osmoregulation, integrity, and pH regulation in Arabidopsis. Proc Natl Acad Sci USA. 111(20):7480–7485. 24794527 10.1073/pnas.1323899111 PMC4034250

[msab096-B54] Le Comber SC, Ainouche ML, Kovarik A, Leitch AR. 2010. Making a functional diploid: from polysomic to disomic inheritance. New Phytol. 186(1):113–122. 20028473 10.1111/j.1469-8137.2009.03117.x

[msab096-B55] Li H, Durbin R. 2009. Fast and accurate short read alignment with Burrows–Wheeler transform. Bioinformatics 25(14):1754–1760. 19451168 10.1093/bioinformatics/btp324 PMC2705234

[msab096-B56] Li H, Handsaker B, Wysoker A, Fennell T, Ruan J, Homer N, Marth G, Abecasis G, Durbin R, 2009. The Sequence Alignment/Map format and SAMtools. Bioinformatics 25(16):2078–2079. 19505943 10.1093/bioinformatics/btp352 PMC2723002

[msab096-B57] Liu CM, Meinke DW. 1998. The titan mutants of Arabidopsis are disrupted in mitosis and cell cycle control during seed development. Plant J. 16(1):21–31. 9807824 10.1046/j.1365-313x.1998.00268.x

[msab096-B58] Lu X, Liu X, An L, Zhang W, Sun J, Pei H, Meng H, Fan Y, Zhang C. 2008. The Arabidopsis MutS homolog AtMSH5 is required for normal meiosis. Cell Res. 18(5):589–599. 18379590 10.1038/cr.2008.44

[msab096-B59] Mandáková T, Marhold K, Lysak MA. 2014. The widespread crucifer species *Cardamine flexuosa* is an allotetraploid with a conserved subgenomic structure. New Phytol. 201(3):982–992. 24400905 10.1111/nph.12567

[msab096-B60] Marburger S, Monnahan P, Seear PJ, Martin SH, Koch J, Paajanen P, Bohutínská M, Higgins JD, Schmickl R, Yant L. 2019. Interspecific introgression mediates adaptation to whole genome duplication. Nat Commun. 10: 5218. 31740675 10.1038/s41467-019-13159-5 PMC6861236

[msab096-B61] Marçais G, Delcher AL, Phillippy AM, Coston R, Salzberg SL, Zimin A. 2018. MUMmer4: a fast and versatile genome alignment system. PLoS Comput Biol. 14(1):e1005944. 10.1371/journal.pcbi.1005944 PMC5802927 29373581

[msab096-B62] Marhold K, Huthmann M, Hurka H. 2002. Evolutionary history of the polyploid complex of *Cardamine amara* (Brassicaceae): Isozyme evidence. Plant Syst Evol. 233(1–2):15–28.

[msab096-B63] Martin A, Orgogozo V. 2013. The loci of repeated evolution: a catalog of genetic hotspots of phenotypic variation. Evolution 67(5):1235–1250. 23617905 10.1111/evo.12081

[msab096-B64] Martin M. 2011. Cutadapt removes adapter sequences from high-throughput sequencing reads. Embnet J. 17(1):10.

[msab096-B65] Mason AS, Pires JC. 2015. Unreduced gametes: meiotic mishap or evolutionary mechanism? Trends Genet. 31(1):5–10. 25445549 10.1016/j.tig.2014.09.011

[msab096-B66] McKenna A, Hanna M, Banks E, Sivachenko A, Cibulskis K, Kernytsky A, Garimella K, Altshuler D, Gabriel S, Daly M, et al 2010. The genome analysis toolkit: a MapReduce framework for analyzing next-generation DNA sequencing data. Genome Res. 20(9):1297–1303. 20644199 10.1101/gr.107524.110 PMC2928508

[msab096-B67] Meyers BC, Morgante M, Michelmore RW. 2002. TIR-X and TIR-NBS proteins: two new families related to disease resistance TIR-NBS-LRR proteins encoded in Arabidopsis and other plant genomes. Plant J. 32(1):77–92. 12366802 10.1046/j.1365-313x.2002.01404.x

[msab096-B68] Molina-Henao YF, Hopkins R. 2019. Autopolyploid lineage shows climatic niche expansion but not divergence in *Arabidopsis arenosa*. Am J Bot. 106(1):61–70. 30609009 10.1002/ajb2.1212

[msab096-B69] Monnahan P, Kolář F, Baduel P, Sailer C, Koch J, Horvath R, Laenen B, Schmickl R, Paajanen P, Šrámková G, et al 2019. Pervasive population genomic consequences of genome duplication in *Arabidopsis arenosa*. Nat Ecol Evol. 3(3):457–468. 30804518 10.1038/s41559-019-0807-4

[msab096-B70] Morgan C, Zhang H, Henry CE, Franklin CFH, Bomblies K. 2020. Derived alleles of two axis proteins affect meiotic traits in autotetraploid *Arabidopsis arenosa*. Proc Natl Acad Sci USA. 117(16):8980–8988. 32273390 10.1073/pnas.1919459117 PMC7183234

[msab096-B71] Narasimhan V, Danecek P, Scally A, Xue Y, Tyler-Smith C, Durbin R. 2016. BCFtools/RoH: a hidden Markov model approach for detecting autozygosity from next-generation sequencing data. Bioinformatics 32(11):1749–1751. 26826718 10.1093/bioinformatics/btw044 PMC4892413

[msab096-B72] Nei M. 1972. Genetic Distance between Populations. Am Nat. 106(949):283–292.

[msab096-B73] Novikova PY, Brennan IG, Booker W, Mahony M, Doughty P, Lemmon AR, Lemmon EM, Dale Roberts J, Yant L, Van de Peer Y, et al 2020. Polyploidy breaks speciation barriers in Australian burrowing frogs Neobatrachus. PLoS Genet. 16(5):e1008769. 32392206 10.1371/journal.pgen.1008769 PMC7259803

[msab096-B74] Ogas J, Kaufmann S, Henderson J, Somerville C. 1999. PICKLE is a CHD3 chromatin-remodeling factor that regulates the transition from embryonic to vegetative development in Arabidopsis. Proc Natl Acad Sci USA. 96(24):13839–13844. 10570159 10.1073/pnas.96.24.13839 PMC24151

[msab096-B75] Panizza S, Tanaka T, Hochwagen A, Eisenhaber F, Nasmyth K. 2000. Pds5 cooperates with cohesion in maintaining sister chromatid cohesion. Curr Biol. 10(24):1557–1564. 11137006 10.1016/s0960-9822(00)00854-x

[msab096-B77] Perruc E, Kinoshita N, Lopez-Molina L. 2007. The role of chromatin-remodeling factor PKL in balancing osmotic stress responses during Arabidopsis seed germination. Plant J. 52(5):927–936. 17892443 10.1111/j.1365-313X.2007.03288.x

[msab096-B78] Pickrell JK, Pritchard JK. 2012. Inference of population splits and mixtures from genome-wide allele frequency data. PLoS Genet. 8(11):e1002967. 23166502 10.1371/journal.pgen.1002967 PMC3499260

[msab096-B79] Preite V, Sailer C, Syllwasschy L, Bray S, Ahmadi H, Krämer U, Yant L. 2019. Convergent evolution in *Arabidopsis halleri* and *Arabidopsis arenosa* on calamine metalliferous soils. Philos Trans R Soc B Biol Sci. 374. 10.1098/rstb.2018.0243 PMC6560266 31154972

[msab096-B80] Quevillon E, Silventoinen V, Pillai S, Harte N, Mulder N, Apweiler R, Lopez R. 2005. InterProScan: protein domains identifier. Nucleic Acids Res. 33(Web Server issue):W116-20. 10.1093/nar/gki442 PMC1160203 15980438

[msab096-B81] R Development Core Team R 2011. R: A Language and Environment for Statistical Computing. Available from: http://www.r-project.org.

[msab096-B83] Rosenberg SC, Corbett KD. 2015. The multifaceted roles of the HOR MA domain in cellular signaling. J Cell Biol. 211(4):745–755. 26598612 10.1083/jcb.201509076 PMC4657174

[msab096-B84] Schmickl R, Koch MA. 2011. Arabidopsis hybrid speciation processes. Proc Natl Acad Sci USA. 108(34):14192–14197. 21825128 10.1073/pnas.1104212108 PMC3161561

[msab096-B85] Schmickl R, Yant L. 2021. Adaptive introgression: how polyploidy reshapes gene flow landscapes. New Phytol. 230(2):457–461. 33454987 10.1111/nph.17204

[msab096-B86] Seear P, France M, Gregory C, Heavens D, Schmickl R, Yant L, Higgins J. 2020. A novel allele of ASY3 promotes meiotic stability in autotetraploid *Arabidopsis lyrata*. PLoS Genet. [Internet] 16(7):e1008900. 32667955 10.1371/journal.pgen.1008900 PMC7392332

[msab096-B87] Selmecki AM, Maruvka YE, Richmond PA, Guillet M, Shoresh N, Sorenson AL, De S, Kishony R, Michor F, Dowell R, et al 2015. Polyploidy can drive rapid adaptation in yeast. Nature 519(7543):349–351. 25731168 10.1038/nature14187 PMC4497379

[msab096-B88] Seppey M, Manni M, Zdobnov EM. 2019. BUSCO: assessing genome assembly and annotation completeness. Methods Mol Biol. 1962:227–245. 31020564 10.1007/978-1-4939-9173-0_14

[msab096-B89] Shaked H, Avivi-Ragolsky N, Levy AA. 2006. Involvement of the arabidopsis SWI2/SNF2 chromatin remodeling gene family in DNA damage response and recombination. Genetics 173(2):985–994. 16547115 10.1534/genetics.105.051664 PMC1526515

[msab096-B90] Siddiqui NU, Stronghill PE, Dengler RE, Hasenkampf CA, Riggs CD. 2003. Mutations in Arabidopsis condensin genes disrupt embryogenesis, meristem organization and segregation of homologous chromosomes during meiosis. Development 130(14):3283–3295. 12783798 10.1242/dev.00542

[msab096-B91] Smedley D, Haider S, Ballester B, Holland R, London D, Thorisson G, Kasprzyk A. 2009. BioMart – biological queries made easy. BMC Genomics 10:22. 19144180 10.1186/1471-2164-10-22 PMC2649164

[msab096-B92] Spalding JB, Lammers PJ. 2004. BLAST Filter and GraphicAlign: rule-based formation and analysis of sets of related DNA and protein sequences. Nucleic Acids Res. 32(1):W22–W36. 10.1093/nar/gkh459 PMC441597 15215343

[msab096-B93] Stanke M, Waack S. 2003. Gene prediction with a hidden Markov model and a new intron submodel. Bioinformatics 19(Suppl 2):ii215–ii225. 14534192 10.1093/bioinformatics/btg1080

[msab096-B94] Stephan AB, Kunz HH, Yang E, Schroeder JI. 2016. Rapid hyperosmotic-induced Ca^2+^ responses in *Arabidopsis thaliana* exhibit sensory potentiation and involvement of plastidial KEA transporters. Proc Natl Acad Sci USA. 113(35):E5242–E5249. 27528686 10.1073/pnas.1519555113 PMC5024618

[msab096-B95] Szklarczyk D, Franceschini A, Wyder S, Forslund K, Heller D, Huerta-Cepas J, Simonovic M, Roth A, Santos A, Tsafou KP, et al 2015. STRING v10: protein-protein interaction networks, integrated over the tree of life. Nucleic Acids Res. 43(Database issue):D447–D452. 25352553 10.1093/nar/gku1003 PMC4383874

[msab096-B96] Szpak M, Mezzavilla M, Ayub Q, Chen Y, Xue Y, Tyler-Smith C. 2018. FineMAV: prioritizing candidate genetic variants driving local adaptations in human populations. Genome Biol. 19(1):5. 10.1186/s13059-017-1380-2 PMC5771147 29343290

[msab096-B97] Takuno S, Ralph P, Swart K, Elshire RJ, Glaubitz JC, Buckler ES, Hufford MB, Ross-Ibarra J. 2015. Independent molecular basis of convergent highland adaptation in maize. Genetics 200(4):1297–1312. 26078279 10.1534/genetics.115.178327 PMC4571994

[msab096-B98] Tamura K, Adachi Y, Chiba K, Oguchi K, Takahashi H. 2002. Identification of Ku70 and Ku80 homologues in *Arabidopsis thaliana*: evidence for a role in the repair of DNA double-strand breaks. Plant J. 29(6):771–781. 12148535 10.1046/j.1365-313x.2002.01258.x

[msab096-B99] Tedder A, Helling M, Pannell JR, Shimizu-Inatsugi R, Kawagoe T, Van Campen J, Sese J, Shimizu KK. 2015. Female sterility associated with increased clonal propagation suggests a unique combination of androdioecy and asexual reproduction in populations of *Cardamine amara* (Brassicaceae). Ann Bot. 115(5):763–776. 25776435 10.1093/aob/mcv006 PMC4373288

[msab096-B100] Tilford CA, Siemers NO. 2009. Gene set enrichment analysis. Methods Mol Biol. 563:99–121. 19597782 10.1007/978-1-60761-175-2_6

[msab096-B101] Van Drunen WE, Husband BC. 2019. Evolutionary associations between polyploidy, clonal reproduction, and perenniality in the angiosperms. New Phytol. 224(3):1266–1277. 31215649 10.1111/nph.15999

[msab096-B103] Vijay N, Bossu CM, Poelstra JW, Weissensteiner MH, Suh A, Kryukov AP, Wolf JBW. 2016. Evolution of heterogeneous genome differentiation across multiple contact zones in a crow species complex. Nat Commun. 7:13195. 27796282 10.1038/ncomms13195 PMC5095515

[msab096-B104] Williams ME, Torabinejad J, Cohick E, Parker K, Drake EJ, Thompson JE, Hortter M, DeWald DB. 2005. Mutations in the Arabidopsis phosphoinositide phosphatase gene SAC9 lead to overaccumulation of PtdIns(4,5)P2 and constitutive expression of the stress-response pathway. Plant Physiol. 138(2):686–700. 15923324 10.1104/pp.105.061317 PMC1150389

[msab096-B105] Wu SY, Culligan K, Lamers M, Hays J. 2003. Dissimilar mispair-recognition spectra of Arabidopsis DNA-mismatch-repair proteins MSH2·MSH6 (MutSα) and MSH2·MSH7 (MutSγ). Nucleic Acids Res. 31(20):6027–6034. 14530450 10.1093/nar/gkg780 PMC219466

[msab096-B106] Yant L, Bomblies K. 2015. Genome management and mismanagement—cell-level opportunities and challenges of whole-genome duplication. Genes Dev. 29(23):2405–2419. 26637526 10.1101/gad.271072.115 PMC4691946

[msab096-B107] Yant L, Hollister JD, Wright KM, Arnold BJ, Higgins JD, Franklin FCH, Bomblies K. 2013. Meiotic adaptation to genome duplication in *Arabidopsis arenosa*. Curr Biol. 23(21):2151–2156. 24139735 10.1016/j.cub.2013.08.059 PMC3859316

[msab096-B108] Yeaman S, Gerstein AC, Hodgins KA, Whitlock MC. 2018. Quantifying how constraints limit the diversity of viable routes to adaptation. PLoS Genet. 14(10):e1007717. 30296265 10.1371/journal.pgen.1007717 PMC6193742

[msab096-B109] Zozomová-Lihová J, Malánová-Krásná I, Vít P, Urfus T, Senko D, Svitok M, Kempa M, Marhold K. 2015. Cytotype distribution patterns, ecological differentiation, and genetic structure in a diploid–tetraploid contact zone of *Cardamine amara*. Am J Bot. 102(8):1380–1395. 26290560 10.3732/ajb.1500052

