# Supplementary material for: Novelty and Convergence in Adaptation to Whole Genome Duplication
Source: Mol Biol Evol. 2021 Mar 30;38(9):3910–24. doi: 10.1093/molbev/msab096 (PMC8382928; doi:10.1093/molbev/msab096)
Supplement: msab096_Supplementary_Data [file msab096_supplementary_data.zip › Bohutinska_Yant_submit_SI.pdf]

Supplemental Information for:

Genomic novelty and process-level convergence in adaptation to whole genome duplication

Magdalena Bohutínská[1,2,*], Mark Alston[3], Patrick Monnahan[3], Terezie Mandáková[4], Sian Bray[5,6], Pirita Paajanen[3], Filip Kolář[1,2,7], and Levi Yant[5,8*]

1. Department of Botany, Faculty of Science, Charles University, Prague, Czech Republic
2. Institute of Botany, The Czech Academy of Sciences, Průhonice, Czech Republic
3. Department of Cell and Developmental Biology, John Innes Centre, Norwich Research Park, Norwich, UK
4. CEITEC – Central European Institute of Technology, and Faculty of Science, Masaryk University, Kamenice, Czech Republic
5. Future Food Beacon of Excellence, University of Nottingham, Nottingham, UK
6. School of Biosciences University of Nottingham, Nottingham, UK
7. Natural History Museum, University of Oslo, Oslo, Norway
8. School of Life Sciences University of Nottingham, Nottingham, UK

***Authors for correspondence:** Levi Yant (levi.yant@nottingham.ac.uk) and Magdalena Bohutínská (magdalena.holcova@natur.cuni.cz)

This PDF file includes:

Supplementary Figures 1 to 4
Supplementary Tables 1 to 9

Other supplementary materials for this manuscript include the following:

One Excel file containing Supplementary Datasets 1 to 3 as three worksheets (Bohutinska_Yant_SupplementaryDatasets.xlsx)

One image file containing Supplementary Figure 5 (FigureS5.pdf)

Supplemental Figures

Supplementary Fig. 1

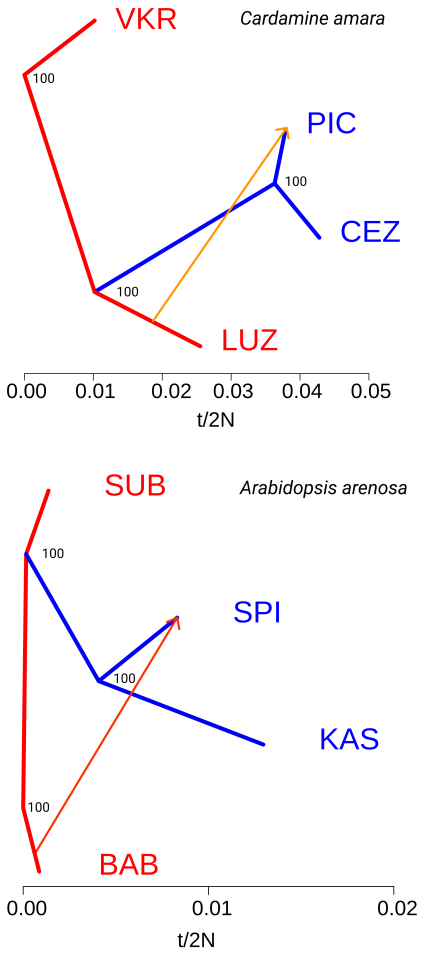

Supplementary Fig. 1: Comparable phylogenetic relationships and migration events between diploid and tetraploid populations of *C. amara* and *A. arenosa* inferred by TreeMix. X-axis shows the drift estimation, corresponding to the number of generations separating the two populations (t), and effective population size (Pickrell and Pritchard 2012). Node labels show bootstrap support and the arrow indicates the most likely migration event (migration weight, which can be interpreted as admixture proportion, = 0.18 and 0.19 for *C. amara* and *A. arenosa*, respectively). Additional migration events did not improve the model likelihood.

Supplementary Fig. 2

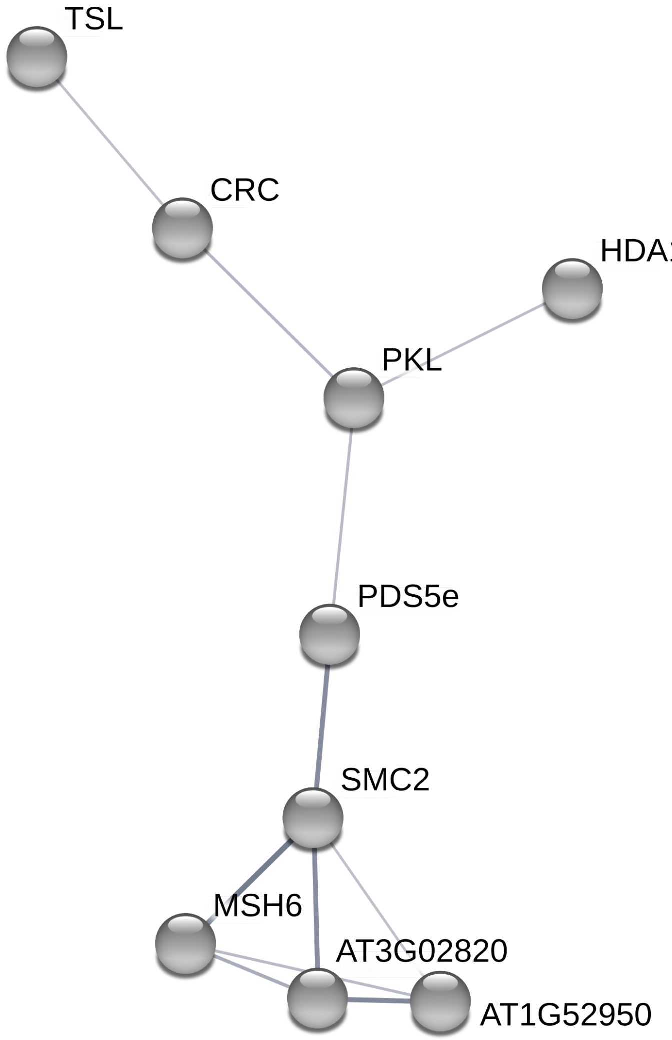

Supplementary Fig. 2: *C. amara* candidate meiosis gene associations as identified by STRING analysis. We used only medium confidence associations and higher (shown as thickness of lines connecting genes).

Supplementary Fig. 3

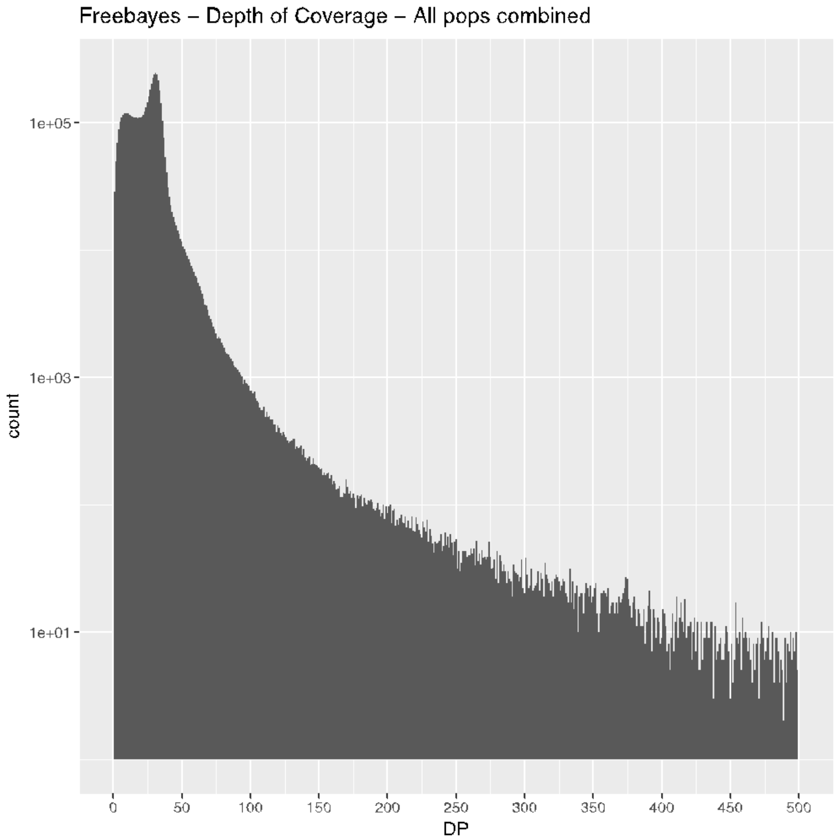

Supplementary Fig. 3: Distribution of read depth over all sequenced samples.

Supplementary Fig. 4

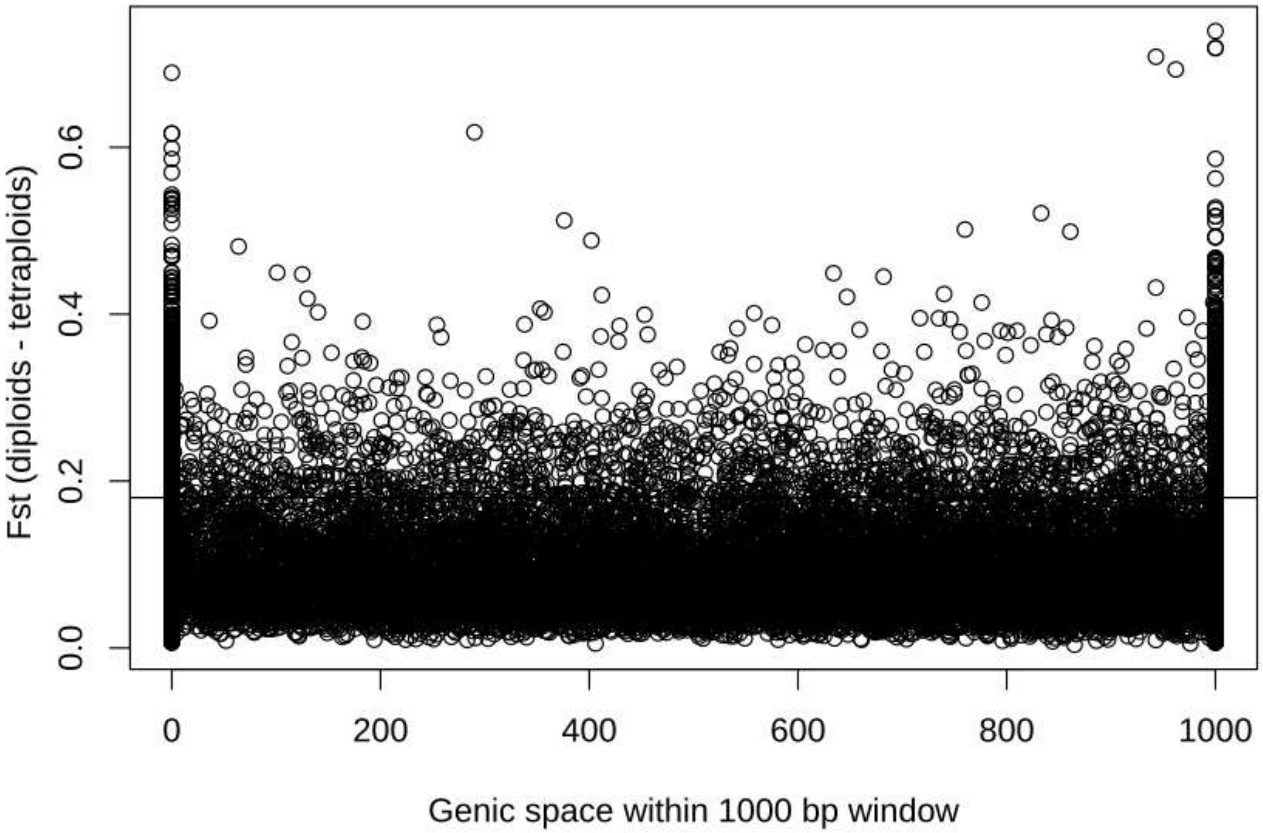

Supplementary Fig. 4: Relationship between the proportion of genic space within a window and Fst.

Supplementary Tables

Supplementary Table 1. GPS coordinates of population localities.

population	ploidy	locality - coordinates
LUZ	2x	50.1384792N, 13.8252669E
VKR	2x	50.4937400N, 14.9075703E
CEZ	4x	48.8873631N, 13.7888903E
PIC	4x	49.2325072N, 13.4175786E

Supplementary Table 2. Mean depth of coverage (MDOC) per pool of individuals from each population.

**population**	**MDOC**
CEZ	64
PIC	85
VKR	92
LUZ	101

Supplementary Table 3. GO terms enriched in *C. amara* WGD candidate genes.

GO.ID	Term	Annotated	Significant	Expected	p-value
GO:0006259	DNA metabolic process	735	23	6.18	6.50E-08
GO:0006468	protein phosphorylation	1287	24	10.82	0.00021
GO:0090502	RNA phosphodiester bond hydrolysis, endo...	162	6	1.36	0.00253
GO:0009617	response to bacterium	510	11	4.29	0.00405
GO:0042742	defense response to bacterium	437	9	3.67	0.01192
GO:0007166	cell surface receptor signaling pathway	237	6	1.99	0.01529
GO:0051276	chromosome organization	720	12	6.05	0.01902
GO:0044036	cell wall macromolecule metabolic proces...	185	5	1.55	0.02026
GO:0046777	protein autophosphorylation	253	6	2.13	0.02038
GO:0009735	response to cytokinin	255	6	2.14	0.02109
GO:0051321	meiotic cell cycle	197	5	1.66	0.02571
GO:0009611	response to wounding	204	5	1.71	0.02928
GO:0051716	cellular response to stimulus	3241	37	27.24	0.03105
GO:0009738	abscisic acid-activated signaling pathwa...	281	6	2.36	0.03186
GO:0048364	root development	527	9	4.43	0.03465
GO:0022622	root system development	528	9	4.44	0.035
GO:0009737	response to abscisic acid	643	10	5.4	0.04579
GO:0097305	response to alcohol	647	10	5.44	0.04739
GO:0009414	response to water deprivation	391	7	3.29	0.04782
GO:0071215	cellular response to abscisic acid stimu...	311	6	2.61	0.04813
GO:0097306	cellular response to alcohol	311	6	2.61	0.04813
GO:0006650	glycerophospholipid metabolic process	164	4	1.38	0.04994

Note: Annotated: # genes in the GO category, Significant: # candidate genes in each category, p-values from Fisher's exact test ('elim' method)

Supplementary Table 4. Targeted search for patterns suggesting directional selection in *C. amara* orthologs of candidate *A. arenosa* meiosis genes.

Protein	A. lyrata ID	C. amara ID	A. thaliana ID	fineMAV (1%)
**PRD3**	AL1G10680	CAg8676	AT1G01690.1	
**SDS**	AL1G26770	CAg1320	AT1G14750.1	
**CYCA2;3**	AL1G27690	CAg1400	AT1G15570.1	1 SNP
**ZYP1b**	AL1G35730	na	AT1G22260.1	
**ASY1**	AL2G25920	CAg28436	AT1G67370.1	
**PDS5b**	AL2G37810	CAg18368	AT1G77600.3	3 SNPs
**ASY3**	AL4G46460	CAg23958	AT2G46980.2	1 SNP
**SCC3**	AL4G47570	CAg23859	AT2G47980.1	
**SYN1**	AL6G15380	CAg4025	AT5G05490.1	
**SMG7**	AL6G30890	CAg5301	AT5G19400.1	
**DYAD**	AL8G25590	na	AT5G51330.1	
**SCC4**	AL8G25600	CAg29925	AT5G51340.1	
**SHOC1**	AL8G26680	CAg10307	AT5G52290.1	

Supplementary Table 5. Chromosome stability scoring of individual diploid (2n = 16) and autotetraploid (2n = 32) plants of *C. amara* at meiotic diakinesis and metaphase I.

Ploidy level	Population	No. spreads scored	No. stable	% stable	No. partly stable	% partly stable	No. partly unstable	% partly unstable	No. unstable	% unstable
2	VKR6	20	6	30	7	35	7	35	0	0
2	VKR8	10	0	0	1	10	2	20	7	70
2	LUZ3	4	1	25	1	25	2	50	0	0
2	LUZ8	30	6	20	12	40	9	30	3	10
2	LUZ10	19	10	52.6	8	42.1	1	5.3	0	0
2	LUZ11	36	28	77.8	8	22.2	0	0	0	0
2	LUZ15	14	8	57.1	6	42.9	0	0	0	0
4	CEZ7	55	0	0	6	10.9	16	29.1	33	60
4	PIC1	45	0	0	8	17.8	23	51.1	14	31.1
4	PIC5	40	6	15	21	52.5	11	27.5	2	5
4	PIC9	13	0	0	5	38.5	8	61.5	0	0
4	PIC11	45	0	0	22	48.9	14	31.1	9	20
4	PIC14	75	3	4	26	34.7	35	46.7	11	14.7
4	PIC18	75	0	0	32	42.7	33	44	10	13.3

Note: In diploids, chromosome spreads with 8 bivalents were scored as "stable meiosis", 7-6 as "partly stable", 5-4 as "partly unstable", and <4 as "unstable". In tetraploids, chromosome spreads with 16 bivalents were scored as "stable meiosis", 14-12 as "partly stable", 10-8 as "partly unstable", and <8 as "unstable". Photos of all spreads scored are shown in Supplementary Figure 5.

Supplementary Table 6. GO terms enriched in *A. arenosa* WGD candidate genes.

GO.ID	Term	Annotated	Significant	Expected	p-value
GO:0000280	nuclear division	244	19	4.13	4.10E-08
GO:1903046	meiotic cell cycle process	162	14	2.74	7.30E-07
GO:0016458	gene silencing	208	15	3.52	2.90E-06
GO:0010608	posttranscriptional regulation of gene e...	289	16	4.9	3.80E-05
GO:0043414	macromolecule methylation	255	14	4.32	0.00012
GO:0051276	chromosome organization	720	30	12.2	0.00021
GO:0006396	RNA processing	929	31	15.74	0.00028
GO:0040029	regulation of gene expression, epigeneti...	183	11	3.1	0.0003
GO:0007275	multicellular organism development	2788	78	47.23	0.00047
GO:0006259	DNA metabolic process	735	25	12.45	0.00082
GO:0019760	glucosinolate metabolic process	155	9	2.63	0.00134
GO:1903047	mitotic cell cycle process	291	13	4.93	0.00148
GO:0048367	shoot system development	951	29	16.11	0.00176
GO:0016071	mRNA metabolic process	583	20	9.88	0.00242
GO:0032502	developmental process	3341	96	56.6	0.00248
GO:0051301	cell division	434	16	7.35	0.0032
GO:0033043	regulation of organelle organization	309	12	5.23	0.00676
GO:0016569	covalent chromatin modification	272	11	4.61	0.00698
GO:0015711	organic anion transport	235	10	3.98	0.00703
GO:1902600	proton transmembrane transport	239	10	4.05	0.00787
GO:0051726	regulation of cell cycle	285	11	4.83	0.00972
GO:0040007	growth	668	20	11.32	0.01049
GO:0031323	regulation of cellular metabolic process	3379	74	57.24	0.01128
GO:0044272	sulfur compound biosynthetic process	180	8	3.05	0.0119
GO:0034660	ncRNA metabolic process	502	16	8.5	0.01223
GO:0009908	flower development	553	17	9.37	0.01385
GO:0009266	response to temperature stimulus	642	19	10.88	0.01388
GO:0003006	developmental process involved in reprod...	1501	37	25.43	0.01433
GO:0000003	reproduction	1864	54	31.58	0.01452
GO:0006417	regulation of translation	187	8	3.17	0.01469

GO:0016570	histone modification	266	10	4.51	0.01577
GO:0080090	regulation of primary metabolic process	3272	71	55.43	0.01592
GO:0034248	regulation of cellular amide metabolic p...	190	8	3.22	0.01602
GO:0090567	reproductive shoot system development	565	17	9.57	0.01673
GO:0009409	response to cold	436	14	7.39	0.01735
GO:0009791	post-embryonic development	1579	38	26.75	0.01855
GO:0048229	gametophyte development	442	14	7.49	0.01926
GO:0050793	regulation of developmental process	758	21	12.84	0.0195
GO:2000112	regulation of cellular macromolecule bio...	2668	59	45.2	0.01961
GO:0051171	regulation of nitrogen compound metaboli...	3207	69	54.33	0.02073
GO:0050794	regulation of cellular process	5341	115	90.48	0.02074
GO:0022414	reproductive process	1855	53	31.42	0.02093
GO:0034470	ncRNA processing	404	13	6.84	0.02106
GO:0006310	DNA recombination	200	8	3.39	0.02109
GO:0010564	regulation of cell cycle process	164	7	2.78	0.02201
GO:0009738	abscisic acid-activated signaling pathwa...	281	10	4.76	0.02214
GO:0048869	cellular developmental process	1007	26	17.06	0.02244
GO:0010556	regulation of macromolecule biosynthetic...	2693	59	45.62	0.02321
GO:0009826	unidimensional cell growth	284	10	4.81	0.02361
GO:0032268	regulation of cellular protein metabolic...	591	17	10.01	0.02456
GO:0048589	developmental growth	458	14	7.76	0.02515
GO:0040008	regulation of growth	335	11	5.68	0.0284
GO:0009657	plastid organization	293	10	4.96	0.02844
GO:0031326	regulation of cellular biosynthetic proc...	2778	60	47.06	0.02854
GO:0009653	anatomical structure morphogenesis	944	24	15.99	0.03221
GO:0051246	regulation of protein metabolic process	611	17	10.35	0.03226
GO:0009889	regulation of biosynthetic process	2800	60	47.43	0.03272
GO:0006364	rRNA processing	261	9	4.42	0.03454
GO:0048608	reproductive structure development	1249	30	21.16	0.03497
GO:0061458	reproductive system development	1251	30	21.19	0.0356
GO:0009658	chloroplast organization	224	8	3.79	0.03768
GO:0032989	cellular component morphogenesis	442	13	7.49	0.03928
GO:0071215	cellular response to abscisic acid stimu...	311	10	5.27	0.04013
GO:0097306	cellular response to alcohol	311	10	5.27	0.04013
GO:0006520	cellular amino acid metabolic process	490	14	8.3	0.04079

GO:0051252	regulation of RNA metabolic process	2523	54	42.74	0.04263
GO:0098655	cation transmembrane transport	474	18	8.03	0.04418
GO:0019219	regulation of nucleobase-containing comp...	2636	56	44.65	0.04423
GO:0016072	rRNA metabolic process	275	9	4.66	0.04555
GO:0006996	organelle organization	2009	64	34.03	0.04621
GO:0006397	mRNA processing	454	13	7.69	0.04684
GO:0055085	transmembrane transport	1339	36	22.68	0.0476
GO:0098662	inorganic cation transmembrane transport	438	17	7.42	0.04996

Note: Annotated: # genes in the GO category, Significant: # candidate genes in each category, p-values from Fisher's exact test ('elim' method).

Supplementary Table 6. Chromosome stability scoring of individual diploid (2n = 16) and autotetraploid (2n = 32) plants of *C. amara* at meiotic metaphase I.

Ploidy level	Population	No. spreads scored	No. stable	% stable	No. partly stable	% partly stable	No. partly unstable	% partly unstable	No. unstable	% unstable
2	VKR6	20	6	30	7	35	7	35	0	0
2	VKR8	10	0	0	1	10	2	20	7	70
2	LUZ3	4	1	25	1	25	2	50	0	0
2	LUZ8	30	6	20	12	40	9	30	3	10
2	LUZ10	19	10	52.6	8	42.1	1	5.3	0	0
2	LUZ11	36	28	77.8	8	22.2	0	0	0	0
2	LUZ15	14	8	57.1	6	42.9	0	0	0	0
4	CEZ7	55	0	0	6	10.9	16	29.1	33	60
4	PIC1	45	0	0	8	17.8	23	51.1	14	31.1
4	PIC5	40	6	15	21	52.5	11	27.5	2	5
4	PIC9	13	0	0	5	38.5	8	61.5	0	0
4	PIC11	45	0	0	22	48.9	14	31.1	9	20
4	PIC14	75	3	4	26	34.7	35	46.7	11	14.7
4	PIC18	75	0	0	32	42.7	33	44	10	13.3

Note: In diploids, chromosome spreads with 8 bivalents were scored as "stable meiosis", 7-6 as "partly stable", 5-4 as "partly unstable", and <4 as "unstable". In tetraploids, chromosome spreads with 16 bivalents were scored as "stable meiosis", 14-12 as "partly stable", 10-8 as "partly unstable", and <8 as "unstable". Photos of all spreads scored are shown in Figure S4.

Supplementary Table 7. *C. amara* candidate genes that have more than one associated protein among *A. arenosa* candidates.

C. amara candidate	# associated candidates in A. arenosa	associated A. arenosa candidates
AT3G21180	3	AT2G31910, AT1G47600, AT1G12040
AT2G25170	5	AT4G38130, AT5G54260, AT3G54670, AT3G06010, AT1G77600
AT4G00630	4	AT4G18160, AT4G10310, AT5G11800, AT1G80300
AT1G30450	3	AT4G11110, AT4G10310, AT1G16310
AT1G15940	5	AT3G54670, AT3G57060, AT5G05490, AT5G51340, AT1G77600
AT4G02070	4	AT5G05490, AT5G54260, AT3G54670, AT3G57060
AT5G62410	10	AT3G57060, AT3G54670, AT5G05490, AT1G77600, AT1G15570, AT5G51340, AT5G55300, AT5G54260, AT5G53450, AT1G67370
AT4G20900	4	AT5G19400, AT5G51330, AT1G14750, AT1G67370
AT5G59220	3	AT5G52300, AT5G52310, AT1G56130
AT5G60820	3	AT3G58040, AT1G15570, AT3G45460
AT1G02680	2	AT3G52270, AT3G16980
AT1G16460	4	AT1G49540, AT3G60600, AT1G50110, AT2G38025
AT1G30790	2	AT5G44980, AT4G02310
AT4G35890	2	AT4G34110, AT1G22760
AT5G61020	3	AT4G09980, AT1G21580, AT3G20050
AT5G23570	4	AT5G55390, AT5G21150, AT1G31280, AT5G04290
AT1G05630	2	AT1G22620, AT2G26890
AT5G45340	2	AT5G52300, AT5G52310
AT1G61340	3	AT5G61010, AT5G65430, AT1G18710
AT4G00620	5	AT5G14660, AT4G20360, AT3G05190, AT2G03430, AT1G16350
AT5G61070	5	AT5G65430, AT4G38130, AT1G78300, AT5G43990, AT3G06010
AT2G28520	5	AT4G23710, AT5G50200, AT3G09790, AT5G14670, AT1G54370
AT5G61030	3	AT4G37910, AT2G30950, AT5G55920
AT2G22440	2	AT2G43370, AT4G32660
AT1G52950	2	AT5G54260, AT3G54670
AT4G35490	6	AT5G02150, AT5G14660, AT4G39280, AT4G20360, AT3G02330, AT3G17910
AT3G19450	2	AT5G22410, AT2G30490
AT3G03410	2	AT4G26470, AT3G03940
AT1G71100	4	AT2G39730, AT3G57940, AT4G18900, AT5G49030
AT3G58990	7	AT5G23010, AT5G23020, AT5G61420, AT4G38220, AT3G14220, AT3G14210, AT1G16410
AT3G58610	10	AT5G23010, AT5G23020, AT4G20360, AT4G14910, AT4G37910, AT1G10060, AT3G05190, AT1G50110,

		AT1G16350, AT3G20050
AT3G53180	3	AT5G23010, AT5G23020, AT5G40260
AT4G15440	2	AT4G15310, AT3G10490
AT4G19120	2	AT4G37910, AT5G27350
AT5G65260	11	AT3G16650, AT1G14640, AT5G04430, AT3G16980, AT3G52270, AT1G21580, AT2G42610, AT4G34110, AT3G02320, AT1G22760, AT5G19400
AT1G73280	5	AT3G53480, AT5G05500, AT5G35190, AT1G47600, AT1G12040
AT5G60780	2	AT5G50200, AT1G33440
AT2G43610	3	AT5G05500, AT1G47600, AT1G12040
AT3G01160	14	AT3G24080, AT3G57940, AT5G55920, AT5G57120, AT5G65900, AT5G64420, AT4G18900, AT3G02320, AT3G16650, AT1G06720, AT2G40430, AT1G67120, AT1G60170, AT2G31660
AT5G63450	3	AT4G32800, AT5G52400, AT4G15310
AT1G35730	2	AT3G18830, AT5G03790
AT4G34450	3	AT5G14670, AT4G38200, AT1G79990
AT3G09925	6	AT5G05500, AT5G22410, AT5G35190, AT3G53480, AT1G12040, AT1G47600
AT3G24240	7	AT3G53480, AT5G05500, AT4G28410, AT5G35190, AT5G22410, AT1G47600, AT1G12040
AT5G02960	12	AT5G05470, AT5G19400, AT5G14660, AT1G06720, AT4G34110, AT1G22760, AT4G20360, AT1G22270, AT3G57940, AT1G79915, AT5G02150, AT3G09790
AT3G02820	3	AT5G55300, AT3G54670, AT1G79890
AT4G32285	2	AT1G79990, AT1G79890
AT2G42740	14	AT4G20360, AT4G34110, AT5G19400, AT5G05470, AT5G14660, AT5G02150, AT3G20050, AT3G24080, AT3G09790, AT4G39280, AT5G55920, AT1G22760, AT2G40430, AT1G67120
AT5G23190	3	AT5G08250, AT1G02205, AT5G19410
AT5G23575	2	AT3G60600, AT2G01070
AT2G22330	6	AT5G61420, AT5G25980, AT5G23020, AT5G23010, AT3G44310, AT1G47600
AT5G61060	5	AT5G65430, AT4G38130, AT1G78300, AT5G43990, AT3G06010
AT2G01830	2	AT4G10310, AT4G08620
AT5G40480	5	AT5G55490, AT5G64420, AT1G67120, AT1G06720, AT3G57940
AT5G23880	11	AT1G14640, AT3G16650, AT3G52270, AT3G16980, AT5G04430, AT3G05580, AT2G43970, AT1G21580, AT5G04290, AT2G35740, AT1G67120
AT3G01150	8	AT5G04430, AT3G16650, AT3G16980, AT3G52270, AT4G34110, AT1G14640, AT1G22760, AT1G49590
AT1G25380	5	AT3G24080, AT5G11560, AT2G40430, AT3G04460, AT1G06720

Supplementary Table 8. Quality checks of DNA isolated from LUZ.

Input sample QC			Final library QC		
Sample	Input QuBit concentration (ng/ul)	Total input in GEM (ng)	QuBit concentration (ng/ul)	Average fragment size (bp)	Molarity based on QuBit (nM)
C_Luz 23	0.62	0.775	0.98	666	0.68

Supplementary Table 9. Assessment of genome completeness using BUSCO.

BUSCO was run in mode: genome
C:94.7%[S:88.7%,D:6.0%],F:1.0%,M:4.3%,n:956

905 Complete BUSCOs (C)
848 Complete and single-copy BUSCOs (S)
57 Complete and duplicated BUSCOs (D)
10 Fragmented BUSCOs (F)
41 Missing BUSCOs (M)
956 Total BUSCO groups searched

**Assembly metrics**
INPUT
- 90.00 M = READS = number of reads
- 139.50 b = MEAN READ LEN = mean read length after trimming; ideal 140
- 60.30 x = RAW COV = raw coverage; ideal ~56
- 47.43 x = EFFECTIVE COV = effective read coverage; ideal ~42 for nominal 56x
- 82.70 % = READ TWO Q30 = fraction of Q30 bases in read 2; ideal 75-85
- 364.00 b = MEDIAN INSERT = median insert size; ideal 0.35-0.40
- 83.49 % = PROPER PAIRS = fraction of proper read pairs; ideal >= 75
- 1.00 = BARCODE FRACTION = fraction of barcodes used; between 0 and 1
- 225.39 Mb = EST GENOME SIZE = estimated genome size
- 14.91 % = REPETITIVE FRAC = estimated repetitive fraction
- 0.50 % = HIGH AT FRACTION = high AT index
- 44.15 Kb = MOLECULE LEN = weighted mean molecule size
- 174.38 = P10 = molecule count extending 10 kb on both sides
- 242.00 b = HETDIST = mean distance between heterozygous SNPs
- 4.42 % = UNBAR = fraction of reads that are not barcoded
- 84.00 = BARCODE N50 = N50 reads per barcode
- 8.39 % = DUPS = fraction of reads that are duplicates
- 57.16 % = PHASED = nonduplicate and phased reads
- 1.78 K = LONG SCAFFOLDS = number of scaffolds >= 10 kb
- 12.54 Kb = EDGE N50 = N50 edge size
- 49.31 Kb = CONTIG N50 = N50 contig size
- 1.65 Mb = PHASEBLOCK N50 = N50 phase block size
- 1.82 Mb = SCAFFOLD N50 = N50 scaffold size
- 16.61 % = MISSING 10KB = % of base assembly missing from scaffolds >= 10 kb
- 159.53 Mb = ASSEMBLY SIZE = assembly size (only scaffolds >= 10 kb)

Other supplementary material

Supplementary Datasets (separate excel file consisting of three worksheets)

Supplementary Dataset 1. Genes in the top 1% of Fst scores (1000 bp windows) in C. amara. Note: red lines denote six genes which are candidates also in A. arenosa.

Supplementary Dataset 2. Top 1% of amino acid substitutions with the highest fineMAV score.

Supplementary Dataset 3. Genes in the top 1% of Fst scores (1000 bp windows) in A. arenosa.

Supplementary Figure 5. (separate image file)
