# Supplementary material for: Novelty and Convergence in Adaptation to Whole Genome Duplication
Source: Mol Biol Evol. 2021 Mar 30;38(9):3910–24. doi: 10.1093/molbev/msab096 (PMC8382928; doi:10.1093/molbev/msab096)

Suppl. Figure 5. DAPI-stained meiotic (metaphase I) chromosomes of diploid (2n = 16; VKR6, VKR8, LUZ3, LUZ8, LUZ10, LUZ11, LUZ15) and tetraploid (2n = 32; CEZ7, PIC1, PIC5, PIC9, PIC11, PIC14, PIC18) populations of *Cardamine amara*. Scale bars, 10 µm.

VKR6

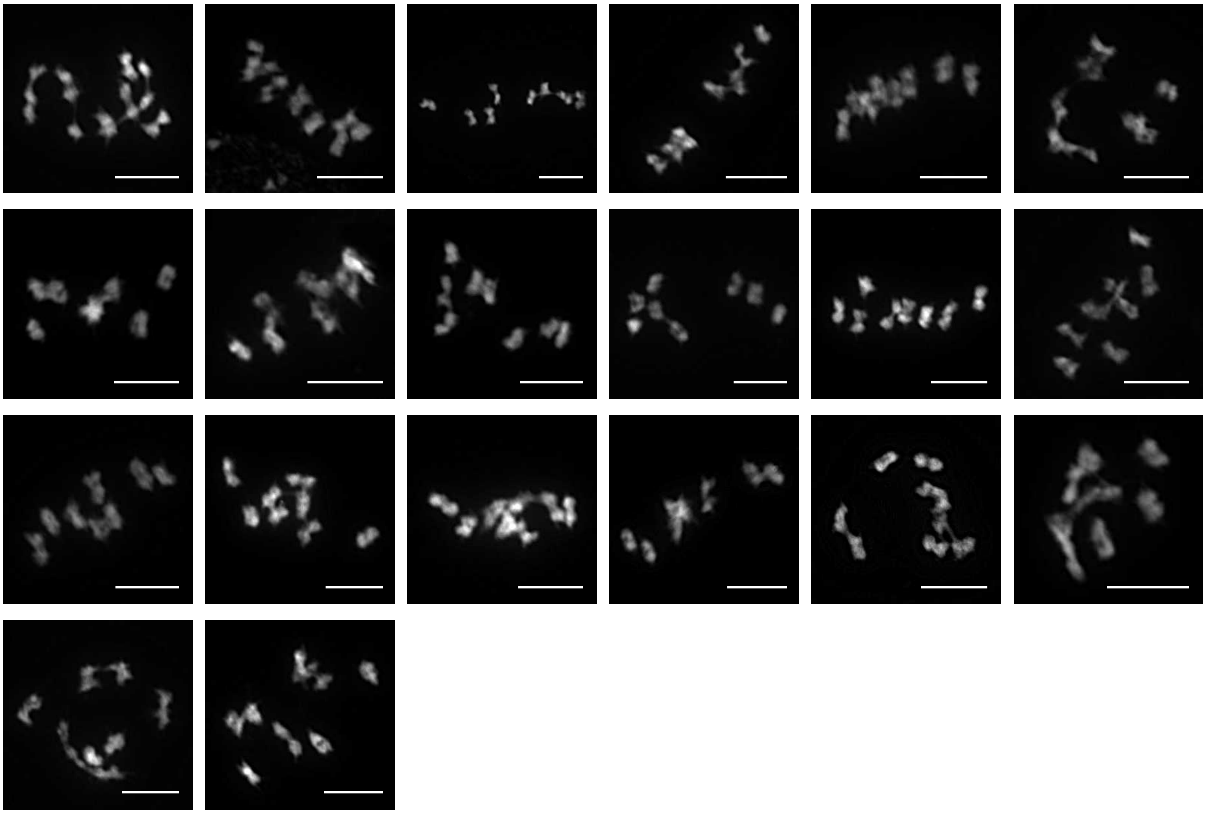

VKR8

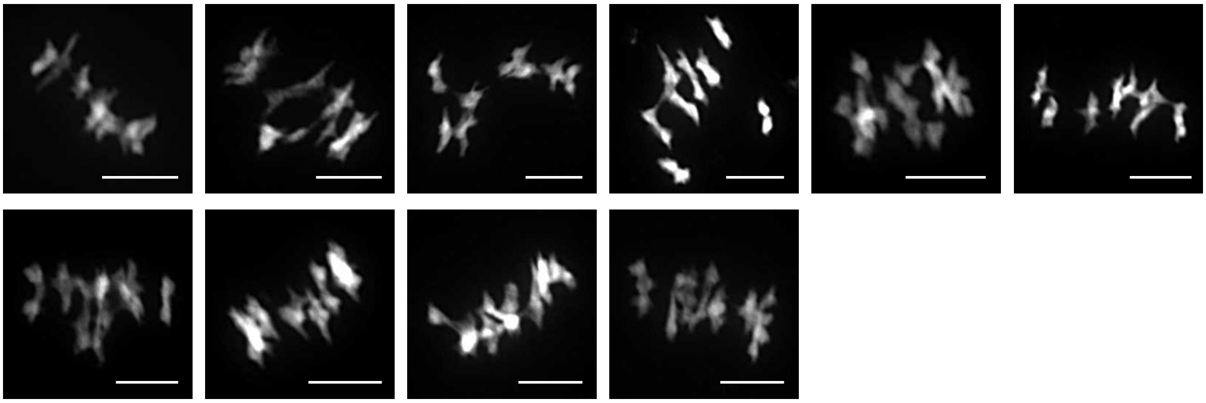

LUZ3

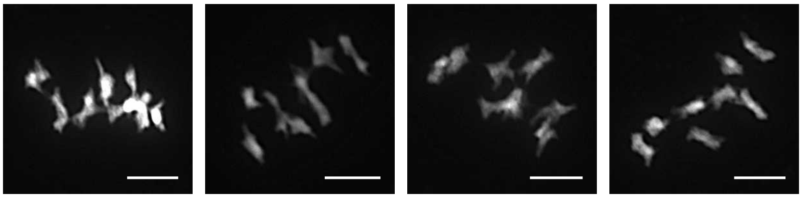

LUZ8

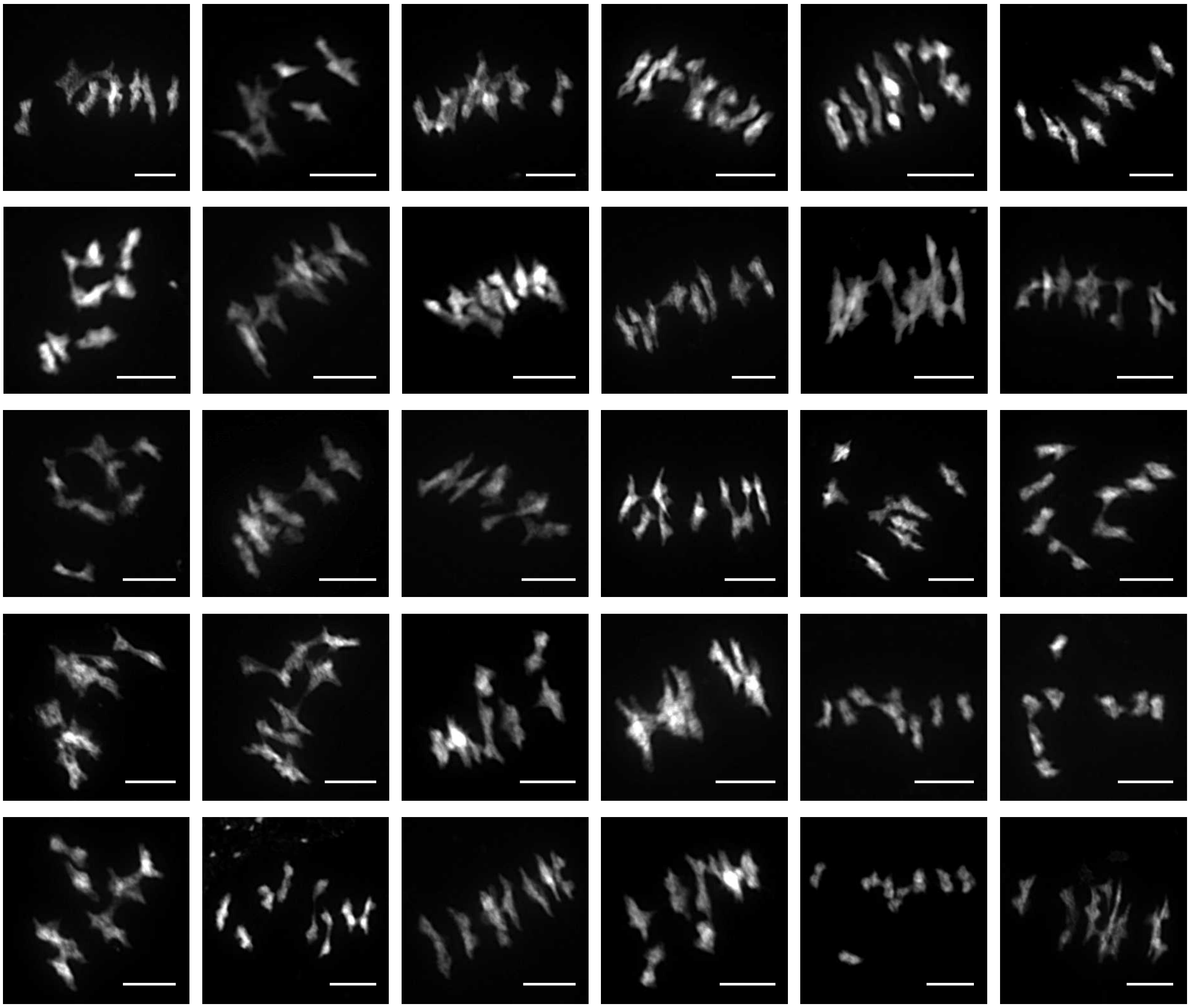

LUZ10

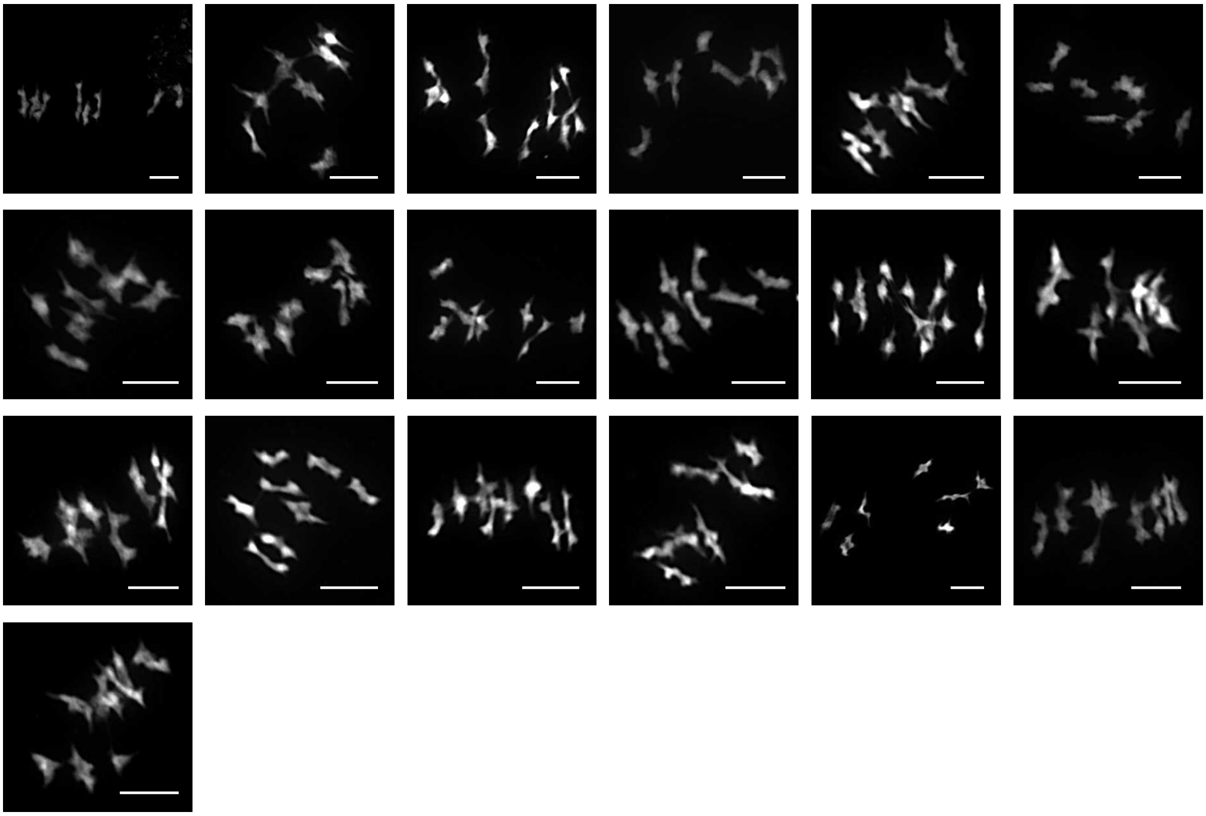

LUZ11

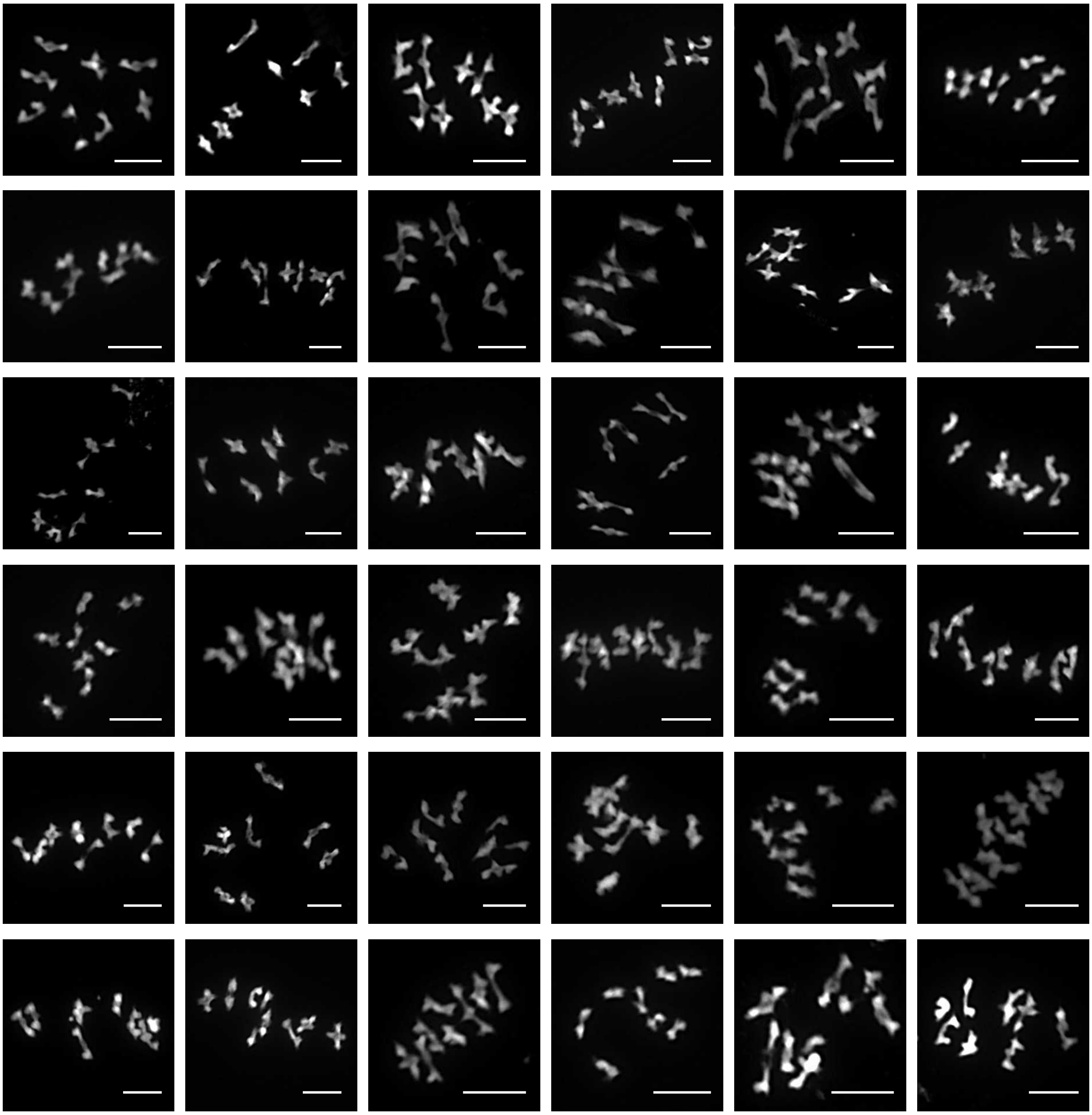

LUZ15

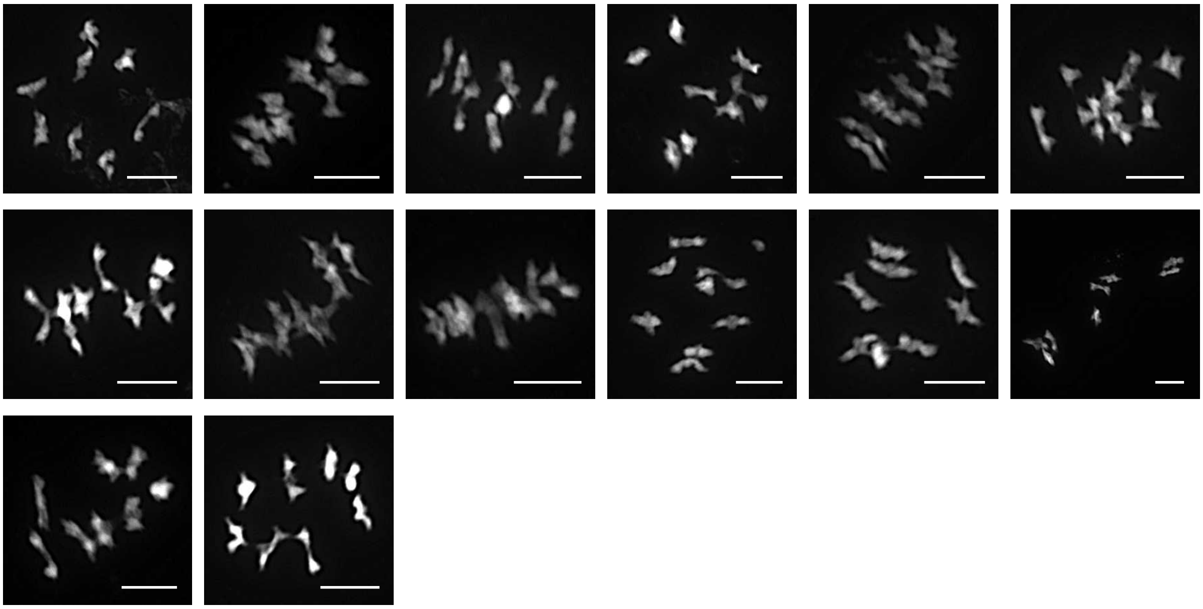

CEZ7

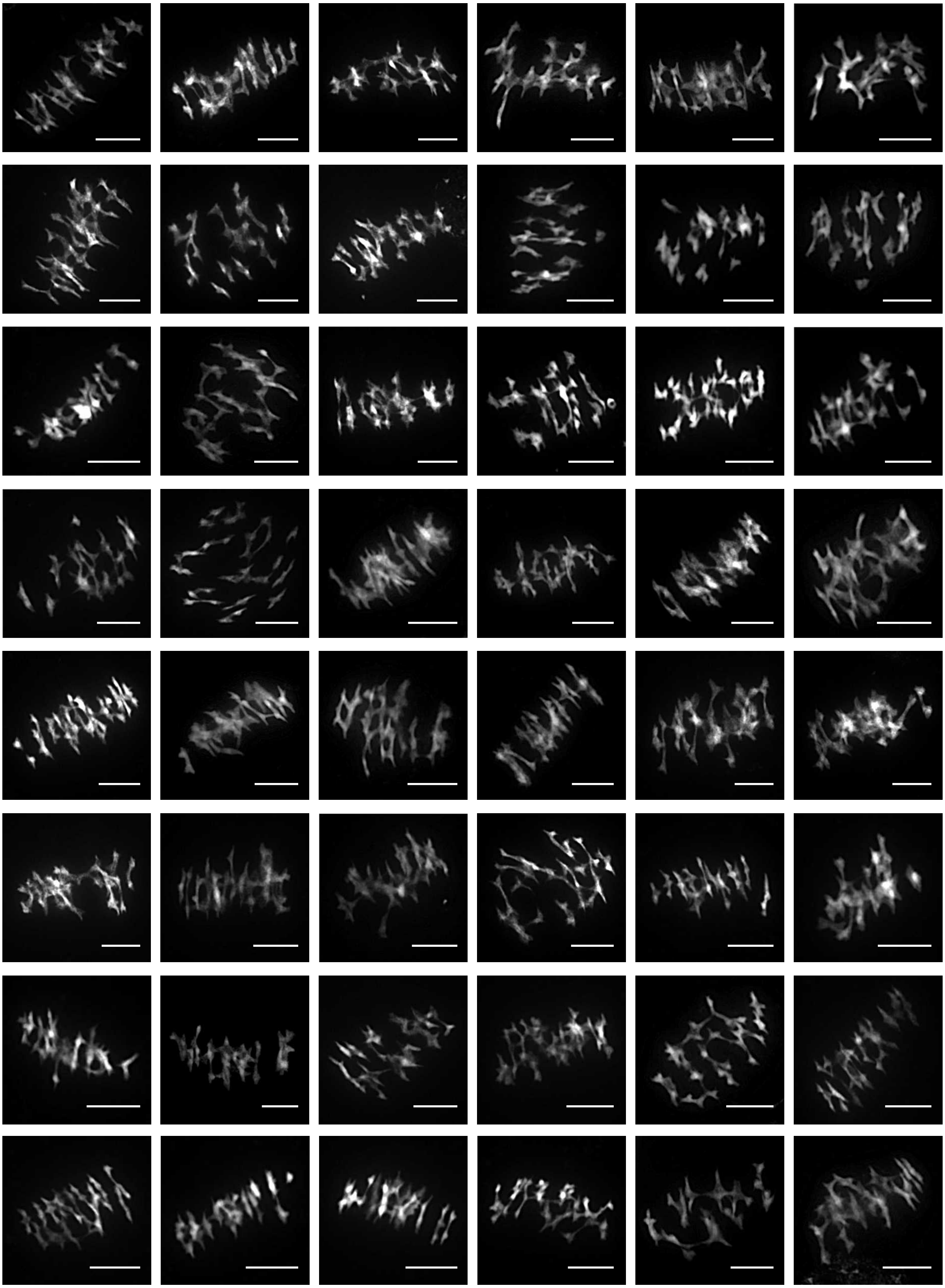

CEZ7 (cont.)

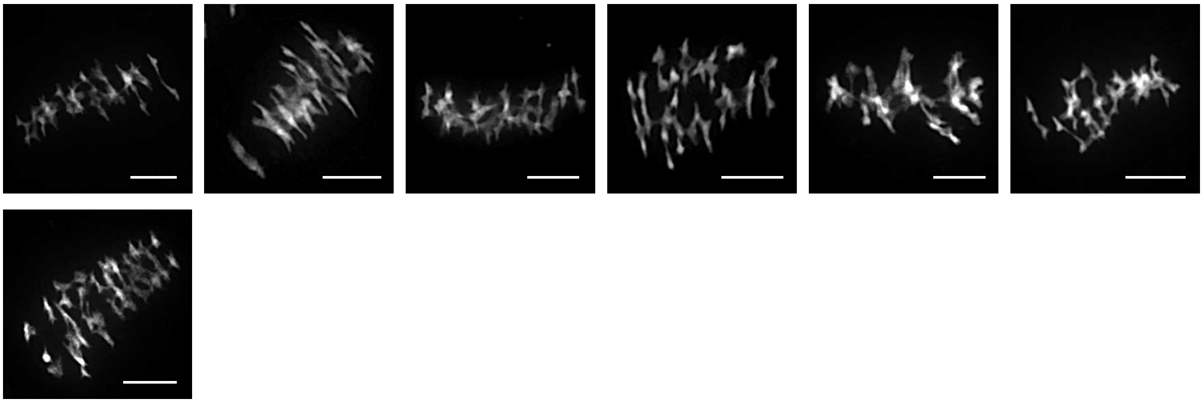

PIC1

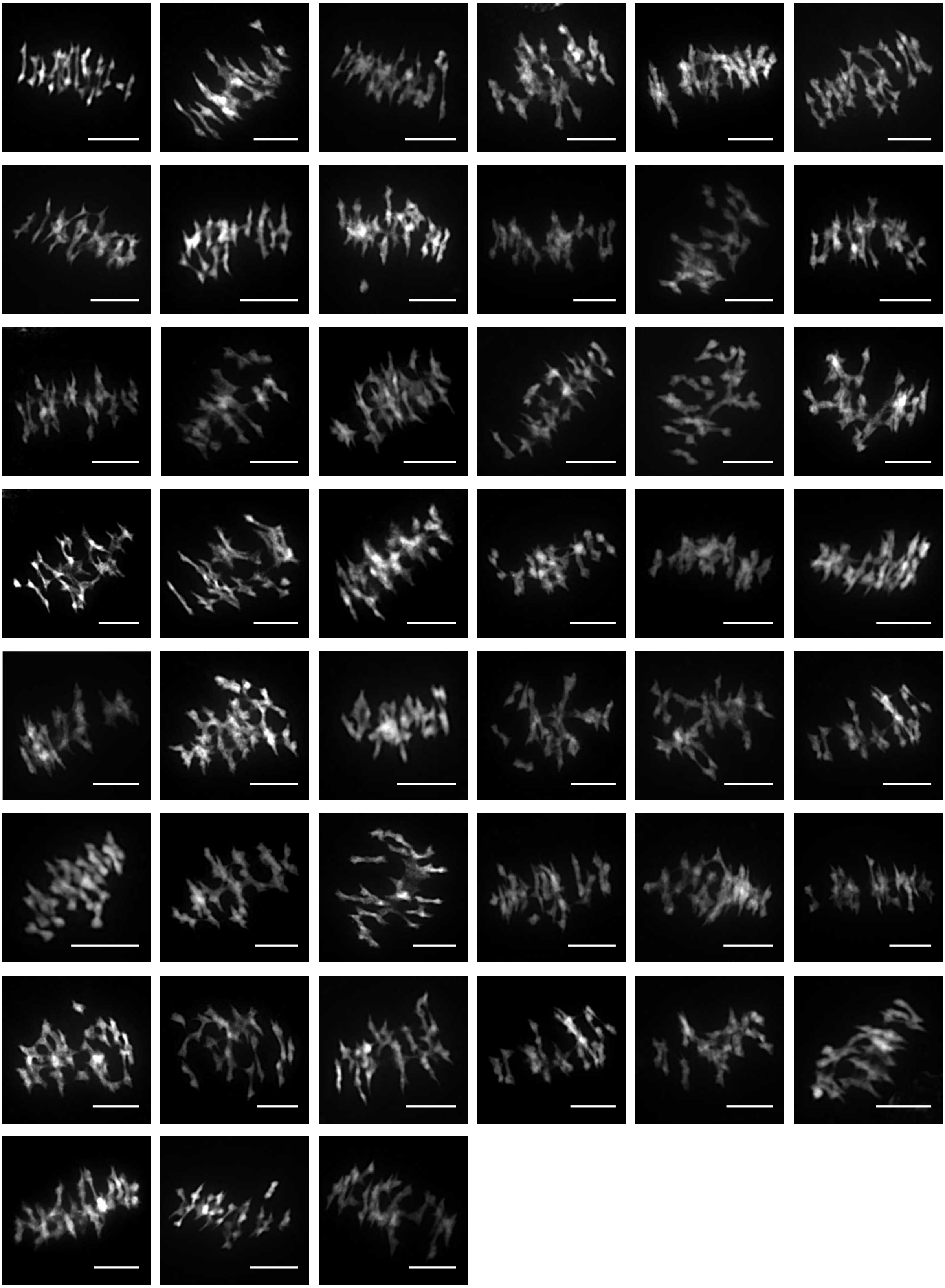

PIC5

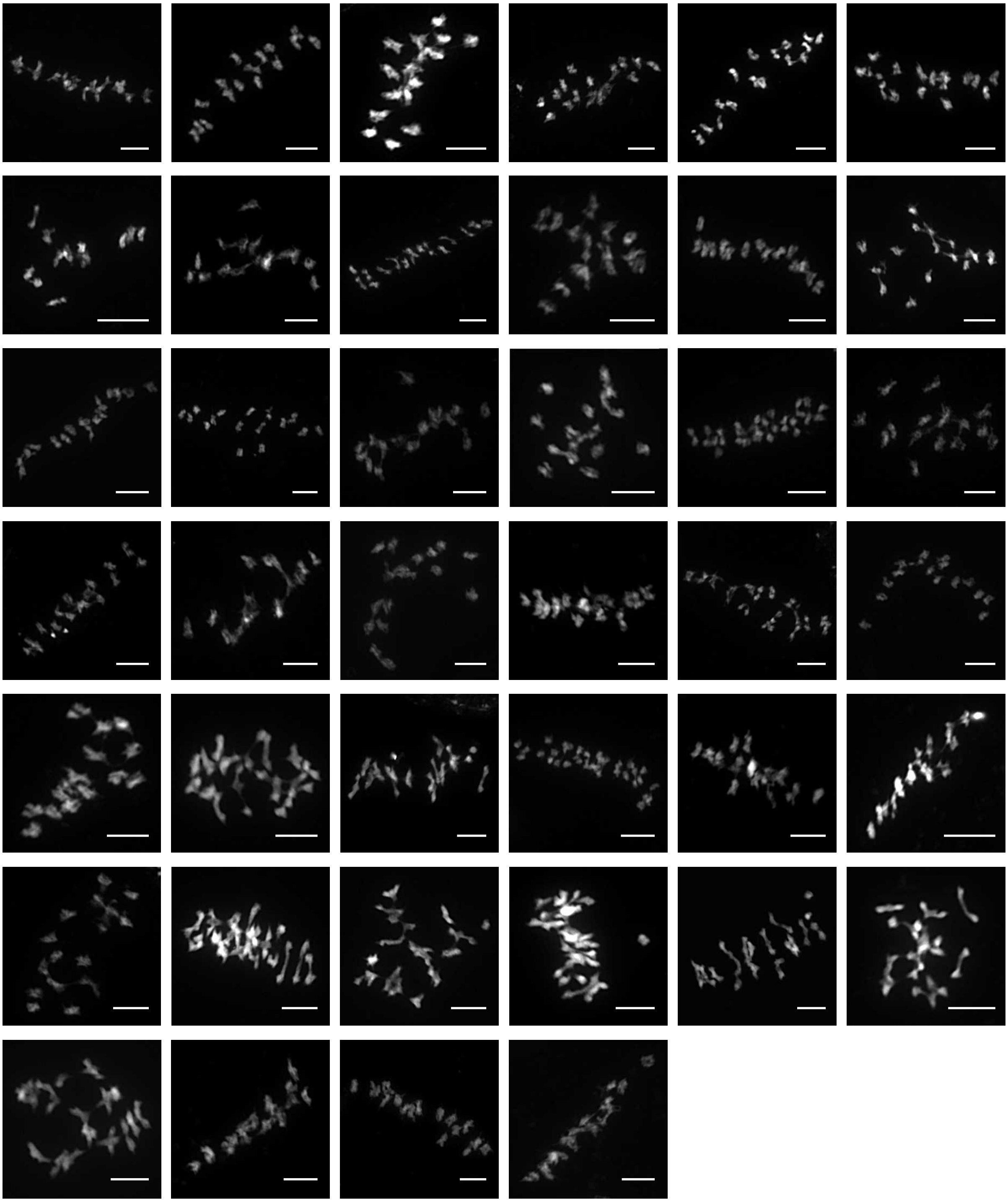

PIC9

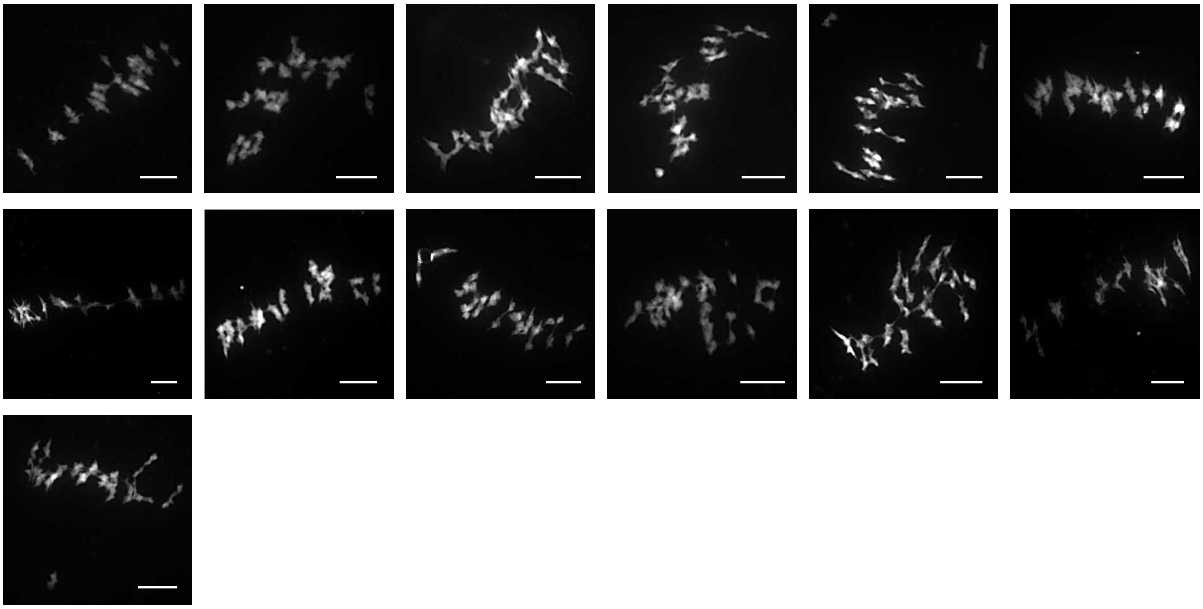

PIC11

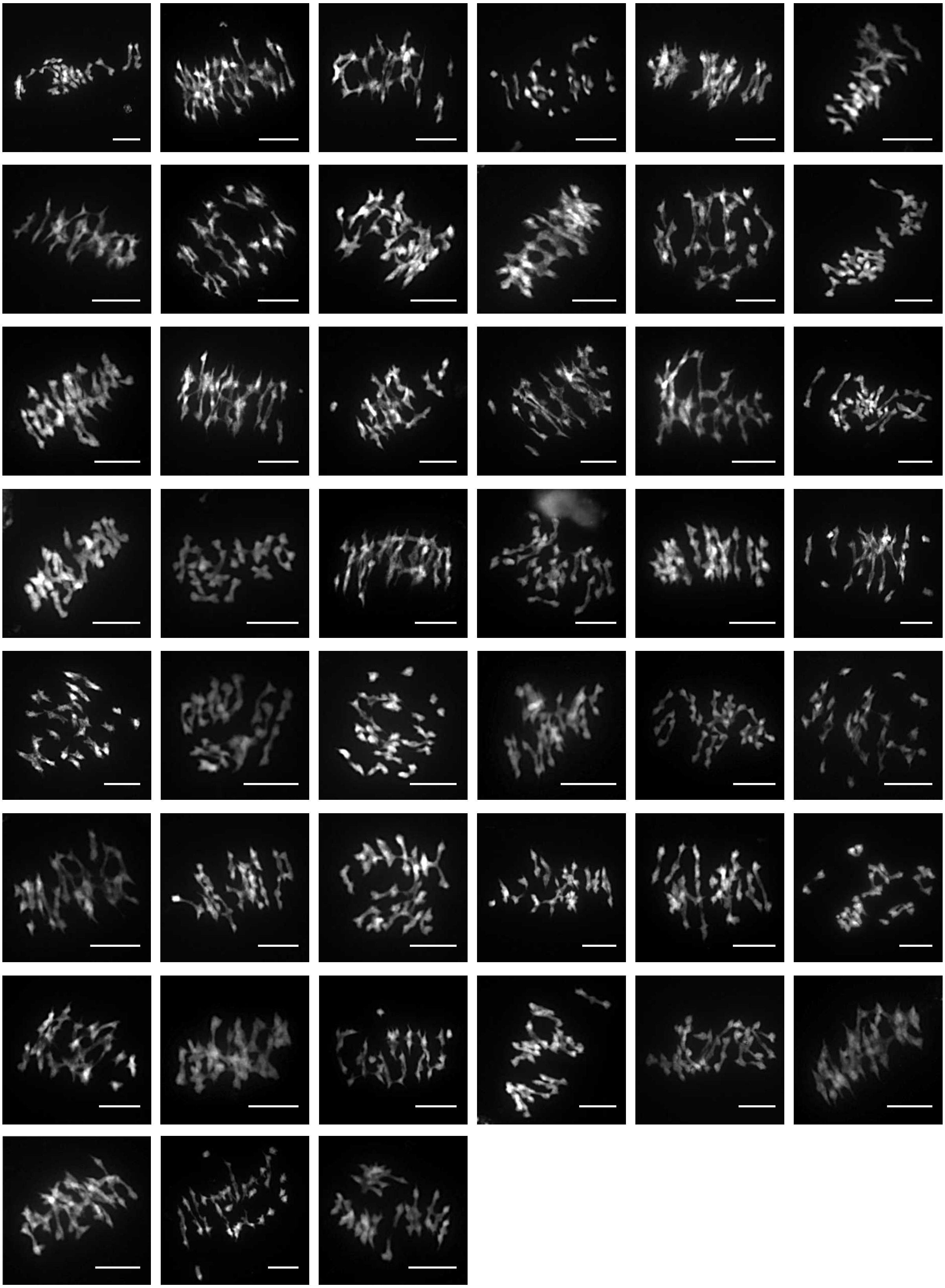

PIC14

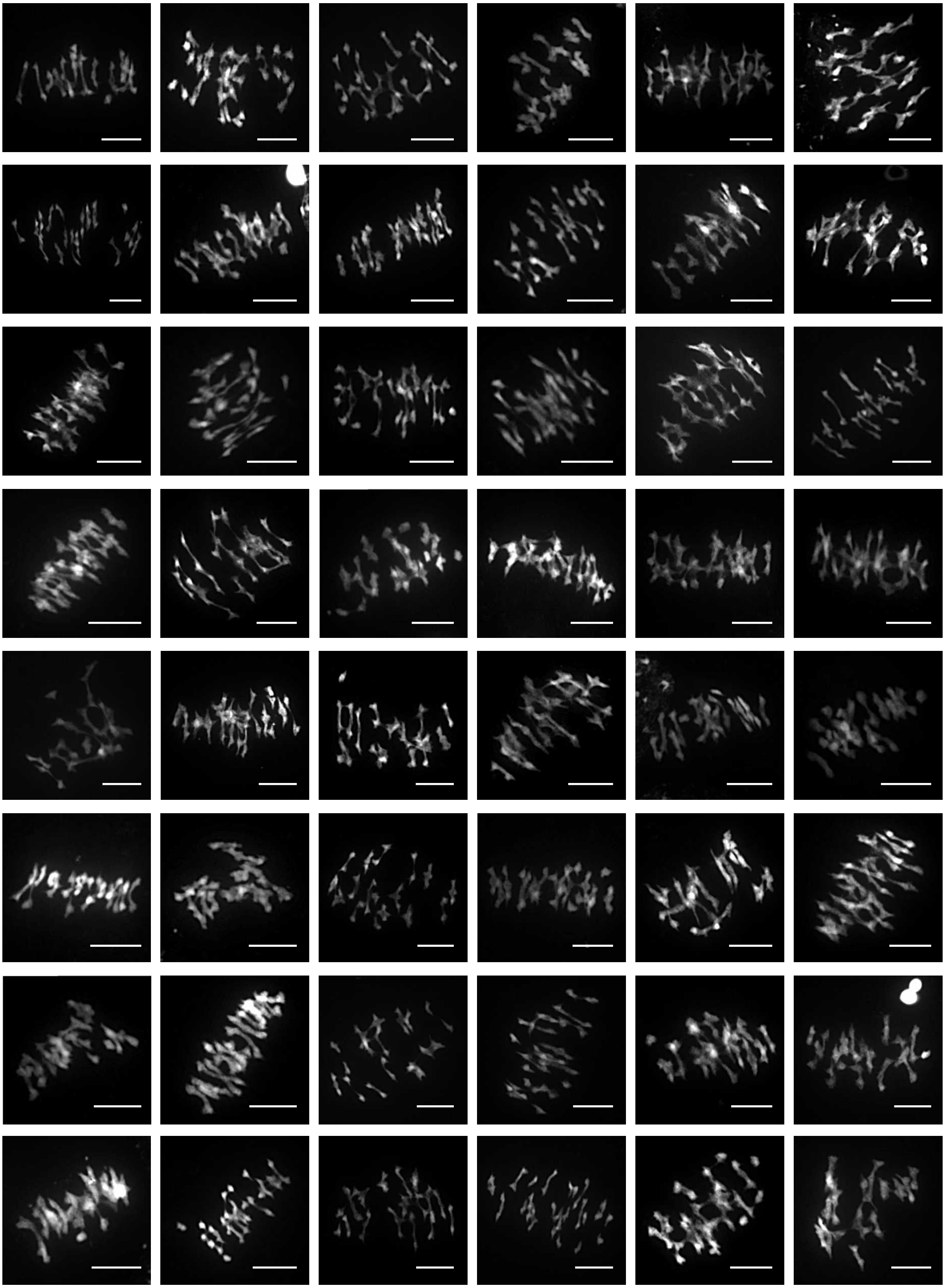

PIC14 (cont.)

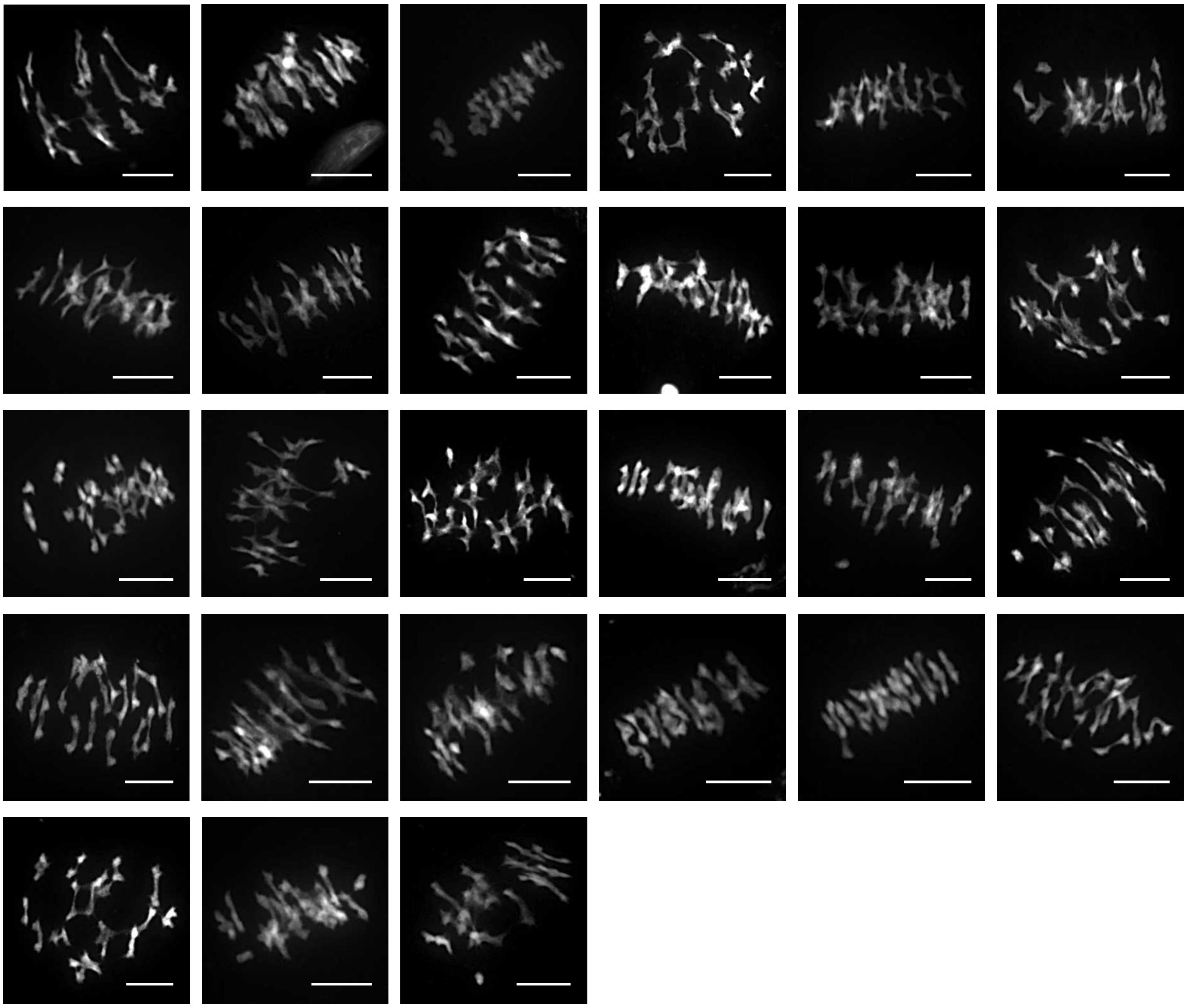

PIC18

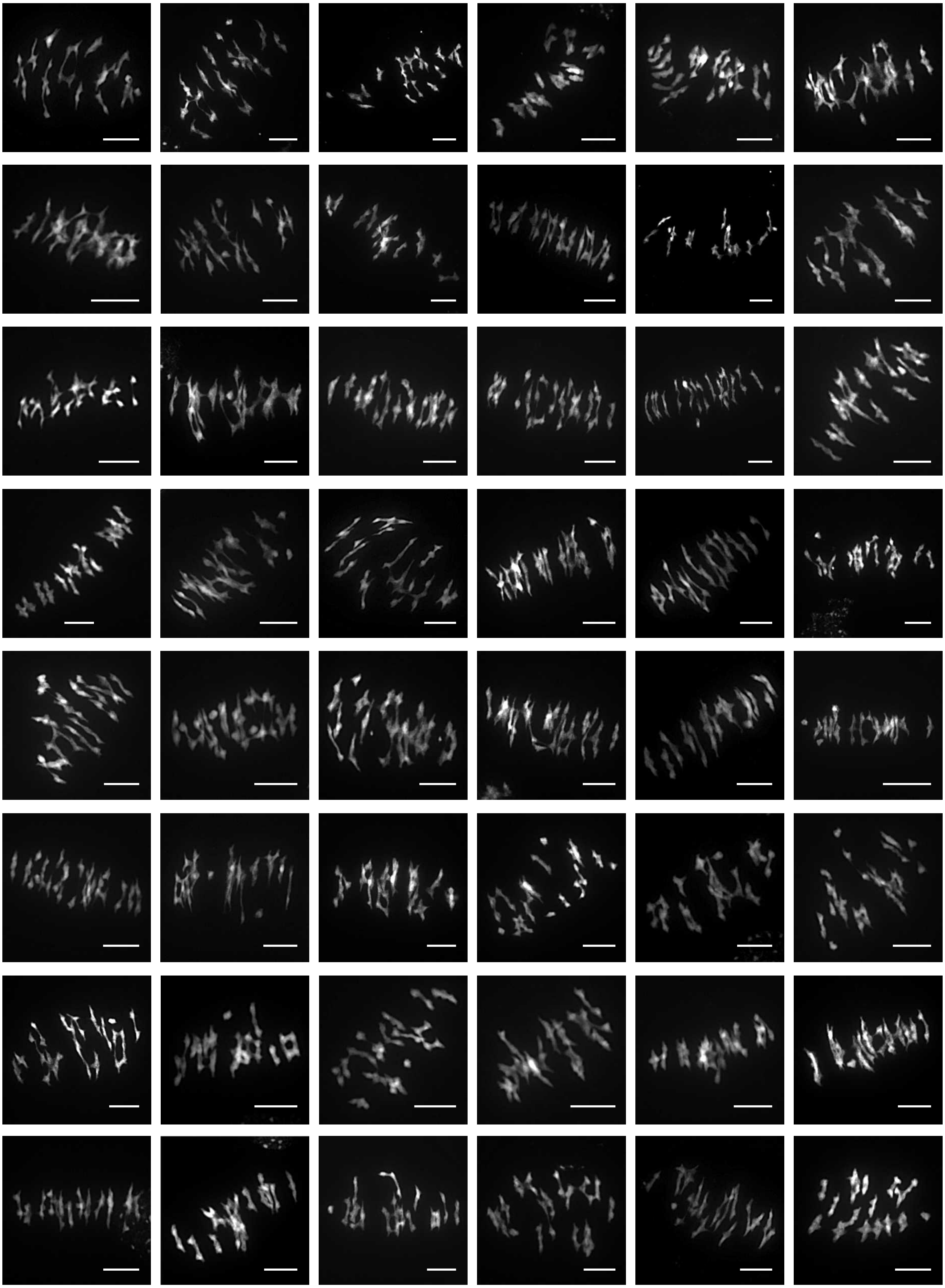

PIC18 (cont.)

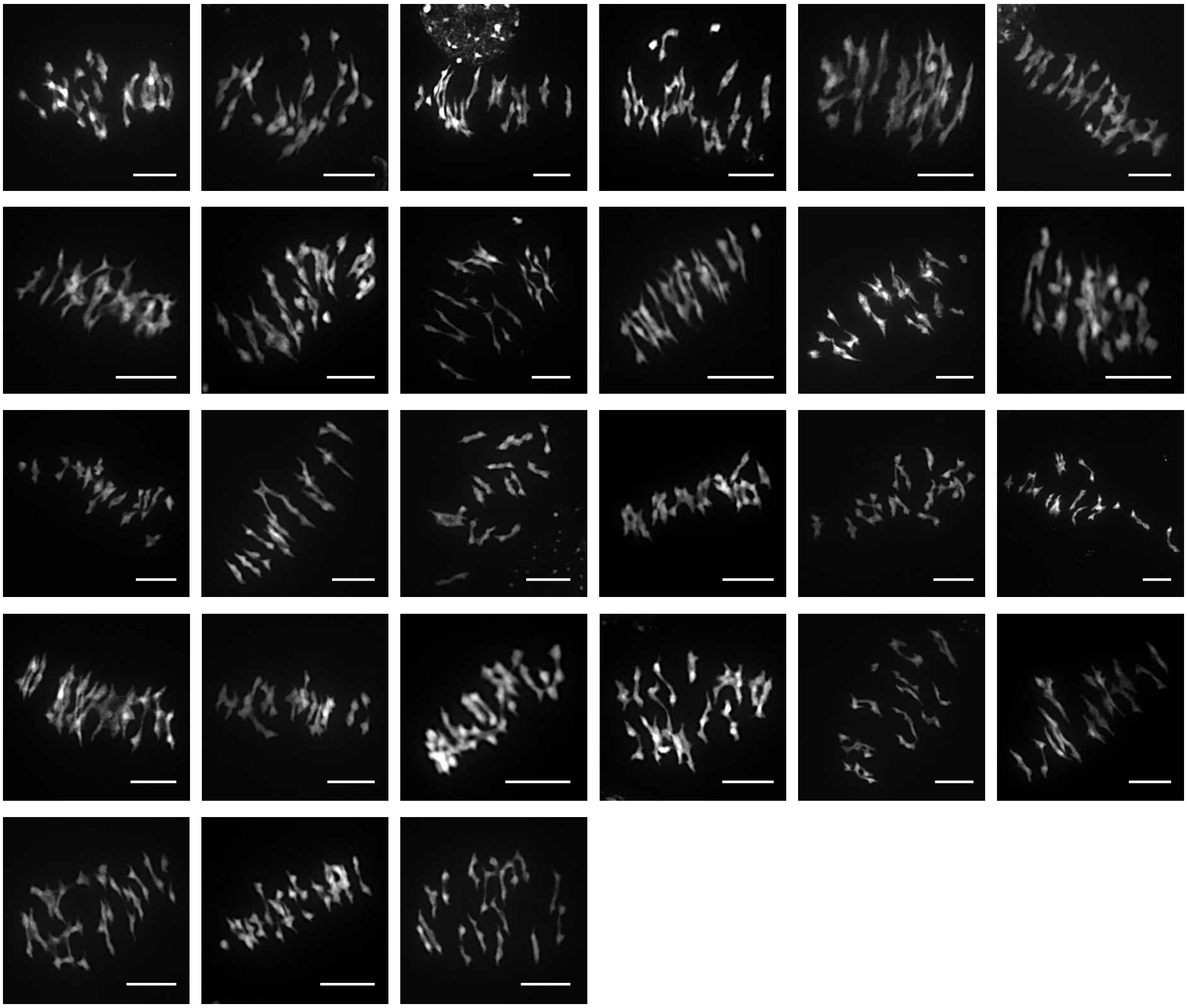

Supplement: msab096_Supplementary_Data [file msab096_supplementary_data.zip › FigureS5.pdf]
